# Treatment of mouse liver slices with cholestatic hepatotoxicants results in down-regulation of Fxr and its target genes

**DOI:** 10.1186/1755-8794-6-39

**Published:** 2013-10-10

**Authors:** Ewa Szalowska, Geert Stoopen, Maria J Groot, Peter JM Hendriksen, Ad ACM Peijnenburg

**Affiliations:** 1 RIKILT - Institute of Food Safety, Wageningen UR, P.O. Box 230, 6700 AE Wageningen, the Netherlands

**Keywords:** Cholestasis, Precision cut liver slices, Alternatives for animal testing, Transcriptome, Fxr (Nr1h4), Biomarkers

## Abstract

**Background:**

Unexpected cholestasis substantially contributes to drug failure in clinical trials. Current models used for safety assessment in drug development do not accurately predict cholestasis in humans. Therefore, it is of relevance to develop new screening models that allow identifying drugs with cholestatic properties.

**Methods:**

We employed mouse precision cut liver slices (PCLS), which were incubated 24 h with two model cholestatic compounds: cyclosporin A (CsA) and chlorpromazine (CPZ). Subsequently, transcriptome analysis using DNA microarrays and q-PCR were performed to identify relevant biological processes and biomarkers. Additionally, histology was carried out and levels of triglycerides (TG) and bile acids (BA) were measured. To verify the ex vivo mouse data, these were compared with publically available human data relevant for cholestasis.

**Results:**

Whole genome gene expression analysis showed that CsA up-regulated pathways related to NF-κB, ER stress and inflammation. Both CsA and CPZ down-regulated processes related to extracellular matrix (ECM) remodelling, BA homeostasis, Fxr signalling, and energy metabolism. The differential expression of a number of characteristic genes (e.g. Abcg5, Abcg8, Klf15, and Baat) could be confirmed by q-PCR. Histology revealed that CsA but not CPZ induced “ballooning” of hepatocytes. No effects on TG and BA levels were observed after incubation of PCLS with CsA and CPZ. A substantial number of processes altered in CsA- and CPZ-treated mouse PCLS ex vivo was also found to be affected in liver biopsies of cholestatic patients.

**Conclusion:**

The present study demonstrated that mouse PCLS can be used as a tool to identify mechanisms of action of cholestatic model compounds. The induction of general stress responses and down-regulated Fxr signalling could play a role in the development of drug induced cholestasis. Importantly, comparative data analysis showed that the ex vivo mouse findings are also relevant for human pathology. Moreover, this work provides a set of genes that are potentially useful to assess drugs for cholestatic properties.

## Background

Drug induced liver injury is a major problem in medical care and pharmacological industry. One example of severe liver damage is cholestasis that comprises approximately 17% of all hepatic adverse drug reactions [1]. Cholestasis results in a dramatic increase in liver and serum bile acids that eventually can lead to liver failure [2]. Several currently used drugs have been shown to induce cholestasis e.g. cyclosporin A (CsA) used as an immunosuppressant is known to induce cholestasis in human, rat and mouse [3,4], and chlorpromazine (CPZ) applied as an antipsychotic was shown to induce cholestasis in both human and rat [5]. It is likely that CPZ can also induce cholestasis in mouse but so far no studies addressing this issue have been reported. Bile acids (BA) are amphipathic molecules synthesized from cholesterol in hepatocytes, which together with phospholipids and cholesterol are actively secreted by the liver as bile. Upon a meal ingestion bile is released into the intestinal lumen to enable absorption of dietary lipids and fat-soluble vitamins [2]. BA levels are tightly controlled because high BA concentrations may become cytotoxic [2].

The nuclear receptor farnesoid X receptor (FXR/NR1H4, in the remaining part of the manuscript FXR symbol will be used) is considered as a master regulator of BA homeostasis. FXR upon BA binding regulates expression of several genes involved in BA homeostasis in the liver [2]. Impairment of FXR signaling and its downstream target genes can result in intrahepatic cholestasis. For example, dysregulation or mutations in genes encoding for bile salt export pump (BSEP), multidrug resistance protein 3 (MDR3) [6], or cholesterol 7a-hydroxylase (CYP7A1) [7] and bile acid-CoA:amino acid N-acyltransferease (BAAT) can evoke cholestasis [8]. Other nuclear receptors (NRs), such as pregnane X receptor (PXR), vitamin D (1,25- dihydroxyvitamin D3) receptor (VDR), and constitutive androstane receptor (CAR) are known to be involved in BA detoxification by regulating expression of genes involved in phase I and II reactions [9]. Moreover, FXR also plays a major role in regulation of lipid and glucose metabolism [2]. Therefore, FXR antagonism does not only induce cholestasis but also leads to increased accumulation of hepatic triglycerides [2]. For that reason, activation of FXR is used in the treatment of cholestasis and non-alcoholic steatohepatitis (NASH) [9].

Consistent with the fact that FXR is involved in both BA and lipid metabolism, drug induced cholestasis can be accompanied with the development of fatty liver diseases [10]. It has also been postulated that drug-induced cholestasis might lead to lipid accumulation as a secondary event of endoplasmatic reticulum (ER) stress causing mitochondrial dysfunction [11]. Additionally, inflammation mediated by Kupffer cells and T cells can contribute to progression of cholestasis [12]. Due to the complexity of BA homeostasis and involvement of different cell types in the development of cholestasis, it is difficult to study this process *in vitro*. Precision cut liver slices (PCLS) are commonly used as an *in vitro/ex vivo* liver model. The major advantage of PCLS is that they contain native liver architecture and all cell types characteristic for the liver *in vivo*[13]. Additionally, it has been shown that pathways mediated by FXR, VDR and PXR are also active in rat and human PCLS, therefore supporting the use of PCLS to study BA metabolism [13]. It has been reported that studies employing toxicogenomics in PCLS can correctly predict the toxicity and pathology observed *in vivo*[14].

The main goal of the present study was to assess whether application of mouse PCLS in combination with whole genome gene expression profiling is an useful approach to identify mechanisms leading to cholestasis. In addition, we aimed to identify genes that could serve as biomarkers to distinguish between cholestasis and other types of hepatotoxicity such as necrosis and steatosis in mouse PCLS. To that end, mouse PCLS were treated for 24 h with two known cholestatic drugs, CsA and CPZ, and subsequently subjected to DNA microarray analysis. Transcriptome analysis was combined with biochemical and histological examinations to allow comparison of the array data with classical parameters of cholestasis.

## Methods

### Chemicals

Cyclosporin A, chlorpromazine, valproic acid, amiodarone, paraquat, and isoniazid were purchased from Sigma (Zwijndrecht, the Netherlands). Williams E medium supplemented with Glutamax, penicillin/ streptomycin (pen/strep), D-Glucose, PBS were obtained from Invitrogen (Bleiswijk, the Netherlands).

### Preparation and culture of liver slices

23-weeks old male C57BL/6 mice were obtained from Harlan (Horst, The Netherlands). Animals were kept for 1 week at a housing temperature of 22°C and at a relative humidity of 30–70%. The lighting cycle was 12-h light and 12-h dark. At the age of 24 weeks animals were sacrificed by an overdose of isoflurane. The treatment protocol was approved by the Ethical Committee for Animal Experiments at Wageningen University.

Immediately after the animals were killed the liver was perfused with PBS and placed in ice-cold Krebs–Henseleit buffer (KHB) (pH 7.4, supplemented with 11 mM glucose). Liver tissue was transported to the laboratory within approximately 30 min and cylindrical liver cores were produced using a surgical biopsy punch with diameter of 5 mm (KAI, SynErgo Europe, Romania). Liver cores were placed in a Krumdieck tissue slicer (Alabama Research and Development, Munford, AL) filled with ice-cold KHB aerated with carbogen and supplemented with 11 mM glucose. Slices with a diameter of 5 mm, a thickness of 0.2 mm and a weight of approximately 6 mg were prepared. Immediately after preparation, slices were transferred into culture plates filled with pre-warmed (37°C) Williams E medium (WEM) supplemented with pen/strep. Three liver slices were pre-cultured in one well of the 6-well plate filled with 4 ml of WEM for one hour with continuous shaking (70 rpm). Incubations were performed in an oxygen controlled incubator (Galaxy 48 R New Brunswick, Nijmegen, the Netherlands) at 80% of oxygen; 5% CO_2_ and the remaining gas volume was filled up to 95% with N_2_. After one hour of pre-incubation, media were removed and replaced with fresh media containing test compounds or appropriate solvents. After incubations, samples were snap-frozen in liquid nitrogen and stored in −80°C for further analysis. Samples dedicated to histology were stored in 4% formaldehyde at room temperature.

### Cytotoxicity analysis (dose selection)

PCLS were exposed to different model compounds inducing cholestasis, steatosis, necrosis, and to compounds that are not hepatotoxic *in vivo*. These compounds were selected based on literature search. As cholestatic model compounds cyclosporin A (CsA), and chlorpromazine (CPZ) were selected. For steatosis valproic acid [15] and amiodarone [16] were selected. As pro-necrotic drugs paraquat [17] and isoniazid [18] were used. To select a non-toxic dose eventually to be used for the gene expression profiling experiments, the following concentrations ranges were tested: cyclosporin A 0–100 μM, chlorpromazine 0–80 μM, valproic acid 0–500 μM, amiodarone 0–100 μM, paraquat 0–10 μM,and isoniazid 0–1000 μM,. CyclosporinA, chlorpromazine, amiodarone, paraquat were dissolved in DMSO, valproic acid was dissolved in ethanol (EtOH), and isoniazid was dissolved in PBS. The compounds were added to the culture medium at a final concentration of 0.1% vol/vol in an appropriate solvent (DMSO, EtOH, or PBS). Slices incubated with the solvents at 0.1% vol/vol served as controls. Toxicity was assessed with standard biochemical assays indicative for slices viability: LDH, ATP, and protein assay. Dose selection experiments were performed in slices obtained from at least two mice (see also Additional file 1: Figure S1, Additional file 2: Figure S2 and Additional file 3: Figure S3).

### ATP and protein measurements

For each ATP and protein measurement a total of three co-cultured slices were placed in 400 μL Cell Lytic MT buffer (Sigma, Zwijndrecht, the Netherlands). In order to compare ATP and protein content in liver slices from different experiments, both ATP and protein were normalized on mass of liver slices. In order to determine the weight of the liver slices, three liver slices were removed from the culture well. Thereafter, the three liver slices were gently held in tweezers against a paper tissue for 2 seconds to let the tissue absorb the remaining medium. Next, the three slices were weighed on a regular laboratory balance and the mass was registered for each individual exposure, which was 18 mg ± 25% for three slices. Slices were homogenized (6500 *g*, 8°C) two times for 15 sec using a tissue homogenizer (Precellys 24 Bertin Technologies, Labmakelaar Benelux B.V. Rotterdam, The Netherlands). To remove cellular debris, the homogenates were centrifuged for 5 min (14000 *g*, 8°C) and the remaining supernatant was divided into two portions of 200 μL. One portion was stored at −80°C for protein measurements and the second 200 μL portion was mixed with 100 μL of ATP lytic buffer (ATPlite, Perkin Elmer, Oosterhout, The Netherlands) for ATP measurements. ATP was measured according to the manufacturer’s description using a microplate reader (Synergy TM HT Multi Detection Microplate Reader, Biotek Instruments Inc, Abcoude, the Netherlands) with settings for luminescence: 590/635 nm, top measurement, and sensitivity 230. ATP determination was performed in technical duplicates and luminescence values were recalculated into μM ATP in total liver slices extracts. ATP concentration was normalized on mass of liver slices used for one ATP extraction.

The protein concentration was determined according to the Bradford method (Protein assay, BioRad, Veenendaal, The Netherlands). Protein samples of 2 μL were diluted 80 times in PBS and measured according to the manufacturer’s instructions. BSA was used as a standard and each measurement was performed in duplicate. The protein content was normalized on mass of liver slices used for protein extraction.

### Lactate dehydrogenase (LDH) assay

Lactate dehydrogenase activity in total tissue homogenate and in the culture medium of each slice was performed according to the manufacturer’s protocol (Cytotoxicity Detection Kit (LDH), Roche Diagnostics, Almere, the Netherlands). For the total LDH activity in slices, three slices were homogenized (6500 *g*, 8°C) twice for 15 sec using a tissue homogenizer (Precellys 24). Thereafter, culture medium or the slice homogenate were diluted 5 fold with PBS and mixed with 100 μL reaction mixture from the kit. After 15 minutes of incubation at room temperature, samples were measured at 490 nm using a Synergy TM HT Multi Detection microplate reader.

### Triglycerides (TG) measurements

Three liver slices cultured together were used to make a homogenate in 300 μL buffer containing 10 mM Tris, 1 mM EDTA (pH 7.4) and 250 mM sucrose. Aliquots of 10 μL of the liver slices homogenates were used in the assay and measurements were performed according to the manufacturer’s protocol (triglycerides liquicolor mono, Instruchemie, Delfzijl, the Netherlands).

### Bile acids measurements

For bile acids measurements, three slices were pooled and placed in 400 μL Cell Lytic MT buffer (Sigma, Zwijndrecht, the Netherlands). Slices were homogenized for two times 15 sec (6500 *g*, 8°C) using a tissue homogenizer (Precellys 24). Thereafter, the homogenates were centrifuged for 5 min (14000 g, 8°C) to remove cellular debris, and the supernatant was used for measurements. Bile acids quantification was performed according to the description in manufacturer’s protocol (Total Bile Acid Assay Kit Bioquant, Gentaur, Brussels, Belgium).

### Histology

Slices were exposed for 24 and 48 hours to 40 μM CsA or 20 uM CPZ. For the 48 hours cultures, after 24 hours the culture medium was replaced with fresh medium. Immediately after incubation, slices were fixed in 4% buffered formaldehyde. After embedding in paraffin, cross-sections were prepared, stained with haematoxylin and eosin (HE) according to Mayer using standard procedures, and examined under a microscope with a 100 x magnification [19]. Fouchet staining for bile acids pigments and Periodic acid-Schiff (PAS) for glycogen were performed according to standard protocols (http://www.ihcworld.com/histology.htm). For each condition slices obtained from 5 mice were analysed. All pictures were taken using 100-fold magnification.

### PCLS exposure (gene expression profiling)

**Figure 1:**
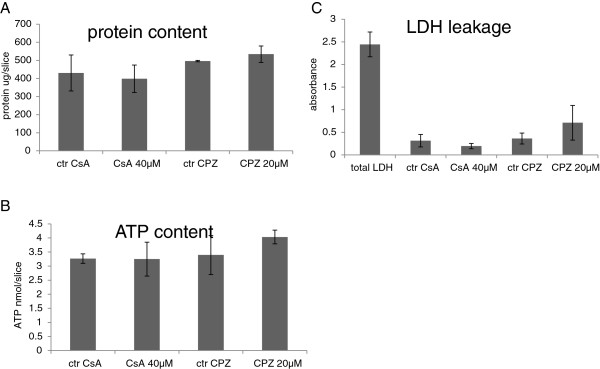
**Biochemical characterization of mouse liver slices in exposure experiments.** Liver slices were incubated for 24 hours with pre-selected concentrations of cyclosporin A (CsA) 40 μM and chlorpromazine (CPZ) 20 μM. Protein content **(A)**, ATP content **(B)** and LDH leakage into medium compared to total LDH content in slices **(C)** were measured to assess liver slices viability. Ctr stands for control. Each point is the mean ± SD of five independent experiments (liver slices were isolated from livers of five mice) and each measurement was performed in technical duplicates. None of the measured parameters were significantly affected.

To obtain RNA for transcriptome analysis, PCLS were cultured at the same conditions as described above. Slices were exposed for 24 hours to one concentration of the tested compounds or controls. Concentrations used in the exposure experiments were pre-selected in the dose selection experiments (cytotoxicity analysis). The highest concentration that did not cause significant toxicity assessed by biochemical assays (LDH, ATP, and protein assay) was selected. The concentrations used in the exposure experiments were as follows; for the cholestatic exposures 40 μM CsA and 20 μM CPZ. For the steatogenic exposures: 200 μM valproic acid, and 50 μM amiodarone. For the necrotic compounds: 5 μM paraquat and 1000 μM isoniazid. Liver slices obtained from five mice were used in five separate experiments in which exposures to toxic compound or vehicle control were performed at the same time. Since dose selection experiments and exposure experiments were performed at different days, ATP, LDH, and protein assays were performed again for the exposure experiments to confirm that the selected doses did also not affect slice viability in these experiments (Figure 1).

### DNA microarray hybridizations

Gene expression analysis in liver slices incubated for 24 hours with the cholestatic, steatogenic, and necrotic compounds as well as the controls was performed on the HT Mouse Genome 430 PM array plate(s) using the Affymetrix GeneTitan system. RNA was extracted from three slices cultured and exposed together using the RNeasy Tissue Mini Kit (Qiagen, Venlo, The Netherlands) according to the manufacturer’s instructions. RNA concentration and purity were assessed spectrometrically using a Nano Drop ND-1000 spectrophotometer (Isogen IJsselstein, The Netherlands) by measuring absorption ratios at 260/280 and 260/230 nm. The integrity of the RNA samples was examined using the Shimadzu MultiNA Bioanalyzer (Tokyo, Japan). Biotin- labelled cRNA was generated from high-quality total RNA with the Affymetrix 3’IVT Express Kit (Affymetrix, Santa Clara, CA, USA) according to the manufacturer’s specifications with an input of 100 ng total RNA. The Agilent Bioanalyzer (Amstelveen, the Netherlands) and Shimadzu MultiNA Bioanalyzer (Tokyo, Japan) were used to evaluate the quality of cRNA in order to confirm if the average fragment size was according to Affymetrix’ specifications. Per sample, 7.5ug cRNA of the obtained biotinylated cRNA samples was fragmented and hybridized in a final concentration of 0.0375 ug /ul on the Affymetrix HT Mouse genome 430 PM array (Affymetrix, Santa Clara, CA, USA. After an automated process of washing and staining by the GeneTitan machine (Affymetrix, Santa Clara, CA, USA) using the Affymetrix HWS kit for Gene Titan, absolute values of expression were calculated from the scanned array using the Affymetrix Command Console v 3.2 software. The data Quality Control was performed in the program Affymetix Expression Console v 1.1 software to determine if all parameters were within quality specifications. The Probe Logarithmic Intensity Error Estimation (PLIER) algorithm method was used for probe summarisation [20].

In order to monitor the sample independent control and the performance of each individual sample during hybridization, hybridizations controls were added to the hybridization mixture. The sample dependent controls such as internal control genes, background values, and average signals were used to determine the biological variation between samples. In conclusion, all the data were within data Quality Control thresholds, according to Affymetrix Expression Console specifications. Non-normalized data in a form of the Cell Intensity File (*.CEL) were re-annotated (EntrezGene htmg430pm_Mm_ENTREZG) and the data were RMA normalized [20,21].

### Gene Set Enrichment Analysis (GSEA)

In order to identify differentially expressed gene sets related to diverse biological functions Gene Set Enrichment Analysis (GSEA) was applied. GSEA was performed using an open access bioinformatics tool (http://www.broadinstitute.org/gsea/index.jsp). In short, this method identifies biologically and functionally related genes affected due to experimental conditions. GSEA applies predefined gene sets that are based on literature or other experiments. Gene sets specify a group of genes specific for a certain biological process, GO ontology, pathway, or user defined groups. GSEA ranks all the genes on their expression ratios between a treatment and the control group and determines whether a particular gene set is significantly enriched at the top or the bottom of the ranked list [22]. Gene sets used in this study were created in an open access bioinformatics tool ANNI http://www.biosemantics.org/index.php?page=ANNI-2-0[23]. ANNI retrieves all the information available on known gene-gene associations present in Medline and can be used, among others, to create gene sets associated with simple queries, for example “inflammation” or “cholestasis”. For the purpose of this study we used several queries related to liver specific and liver non-specific processes. Summary of all the queries used for the creation of “the ANNI gene sets” is given in Additional file 4: Table S1. Genes present in at least five publications indicating an association with the specified queries were included in the ANNI gene sets.

For GSEA, also Gene Expression Omnibus microarray data relevant for human cholestasis were used (GSE46960). These gene expression data were generated in GeneChip® Human Gene 1.0 ST array (Affymetrix, CA) hybridisation experiments using human liver biopsies obtained from 64 infants with biliary atresia, 14 age-matched infants with cholestasis of other origin than biliary atresia, and from 7 deceased-donor healthy children.

### MetaCore pathway analysis

The GSEA report output file informs which gene sets are significantly affected in the analysed groups. Additionally, it informs, which genes significantly contribute to this enrichment (significant genes). These significant genes were used as an input for the MetaCore pathway analysis. The pathway analysis was performed using Functional Ontology Enrichment/ Pathways Maps default option in MetaCore for mouse or human depending whether mouse or human data were analysed, respectively. MetaCore identifies pathways using a default enrichment analysis. The identified pathways were considered as significantly affected by the treatment if p <0.005.

### Biomarker identification

In order to identify biomarkers indicative for the development of cholestasis, significant genes identified in GSEA were subjected to Venn analysis. Genes overlapping between CsA and CPZ treatments with absolute FC ≥1.5 were selected as potential biomarkers. Venn analysis was performed using an open access tool http://bioinfogp.cnb.csic.es/tools/venny/. Subsequently, biomarkers’ expression values (derived from the DNA microarray data) were compared with expression values for the same genes in slices treated with non-cholestatic drugs. In order to do so, the gene expression values were log2 transformed and median centered followed by hierarchical clustering using default options in Genesis (http://genome.tugraz.at/genesisserver/genesisserver_description.shtml). Additionally, the candidate biomarkers were uploaded to another open access bioinformatics tool denoted with Search Tool for the Retrieval of Interacting Genes/Proteins 8.2 (STRING) in order to perform functional clustering. STRING identifies and visualizes functional networks based on known and predicted protein-protein interactions [24]. Functional clustering of genes was performed in STRING using functional clustering function.

### q-PCR

In further studies, expression of ten candidate biomarkers in PCLS was analyzed by q-PCR using the Biorad CFX96 TM Real-Time Detection System (Bio-Rad, Veenendaal, The Netherlands) with the following cycling conditions: 15 min 95°C followed by 40 cycles of 15 s 95°C and 1 min 60°C. Reactions were performed in 10 μl and contained 20 ng cDNA, 1x TaqMan PCR Master Mix (Applied Biosystems, Foster City, CA), and depending on the analysed gene, 250 nM probe and 900 nM primer (for actin b) or 1xTaqMan gene expression assay (for the remaining genes) (Applied Biosystems, foster City, CA). Specific primer set for actinβ (ACTB) was developed with Primer Express 1.5 (Applied Biosystems, Nieuwerkerk aan den IJssel, The Netherlands) and sequences were as follows, probe: TGT CCC TGT ATG CCT CTG GTC GTA CCA C, forward primer: AGC CAT GTA CGT AGC CAT CCA, and reverse primer: TCT CCG GAG TCC ATC ACA ATG. Primers for Kruppel-like factor 15 (Klf15), bile acid-CoenzymeA:amino acid N-acyltransferase (Baat), ATP-binding cassette, sub-family G (WHITE), member 8 (Abcg8), ATP-binding cassette, and sub-family G (WHITE), member 5 (Abcg5), were purchased from Applied Biosystems. The numbers of assays from Applied Biosystems are given in Additional file 5: Table S2. Data were analyzed with SDS 2.0 software (Bio-Rad). For each sample, the RT-PCR reaction was performed in triplicate and the averages of the obtained threshold cycle values (C_T_) were processed for further calculations. To check for contaminating DNA, samples without reverse transcriptase (−RT reaction) were analysed as well. For normalization ACTB was used. Relative expression was calculated with ∆ (∆ (C_T_))-method [25].

### Statistical analysis

Mann–Whitney test was used to calculate differences between controls and slices cultured with the tested compounds for ATP, protein, LDH, TG, and bile acids. The cut off for statistical significance was set at a *p*-value < 0.05.

Mann–Whitney (MW) test was used to determine if there was a significant difference in gene expression assessed by q-PCR in slices exposed to different compounds. A *p*-value <0.05 was considered significant.

## Results

### Exposure of PCLS to CsA and CPZ

In order to identify non-cytotoxic concentrations of the model cholestatic compounds (CsA and CPZ), dose selection exposure experiments were performed. In these experiments, PCLS were incubated for 24 hours with four different concentrations of each drug. The applied concentrations were for CsA 1, 2, 40, and 100 μM and for CPZ 2, 4, 20, and 80 μM. Viability of the PCLS was assessed by using LDH leakage, ATP, and protein content as parameters. On the basis of the results from the viability measurements, the highest concentration that did not evoke significant decrease in viability was selected for subsequent gene expression profiling experiments. The results from the dose selection experiments are presented in Additional file 1: Figure S1. With respect to CPZ, a concentration of 20 μM was chosen for the microarray experiment. Although CsA even at the concentration of 100 μM did not induce a decrease in viability, 40 μM was selected for the microarray experiment since this concentration was also used in other *in vitro* studies [26]. Results from the dose selection experiments are presented in Additional file 1: Figure S1A-C. Since the exposure for gene expression profiling was performed with liver slices from mice other than those used for dose selection, the viability of these slices was tested as well. As shown in Figure 1, it could be confirmed that 40 μM CsA and 20 μM CPZ did not affect the viability of the slices.

### Transcriptome analysis: biological processes and pathway analysis

**Figure 2:**
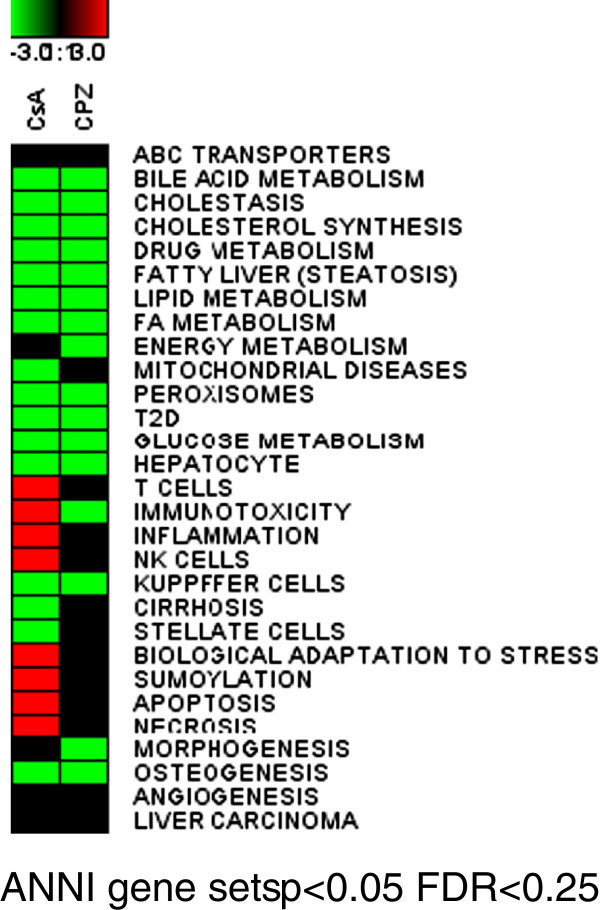
**Effects of cholestatic drugs on gene expression in mouse PCLS.** PCLS obtained from 5 mice were treated with 40 μM cyclosporin A (CsA), 20 μM chlorpromazine (CPZ) or vehicle (DMSO) for 24 hours and subjected to Affymetrix microarray analysis. The biological processes in the heat map correspond to gene sets significantly affected according to GSEA analysis (p < 0.05, FDR < 0.25). Gene sets were obtained using the ANNI text mining tool. Processes that were up-regulated are represented by red color, the down-regulated processes are depicted in green, processes that were unchanged are depicted in black.

Upon treatment of the slices for 24 h with 40 μM CsA or 20 μM CPZ, RNA was isolated and hybridized to DNA microarrays. In order to identify and to compare significantly enriched processes, GSEA was performed using gene sets made in the text-mining tool ANNI. The gene sets (listed in Additional file 4: Table S1) were related to diverse hepatic (e.g. lipid and glucose metabolism, inflammation, bile acid metabolism) and non- hepatic biological processes (e.g. adipogenesis, morphogenesis, tight junctions). As indicated in Figure 2, CsA and CPZ affected several of the gene sets/processes (p < 0.05, FDR < 0.25). Both compounds down-regulated processes related to cholesterol, bile acids, lipid, and glucose metabolism. The gene set related to Kupffer cells was also down-regulated by both compounds. CsA, but not CPZ, up-regulated processes related to inflammation (such as T cells, immunotoxicity, inflammation, and natural killer cells) and molecular processes involved in cell death (apoptosis, necrosis) and stress response (biological adaptation to stress, and sumoylation). Moreover, CsA down-regulated processes related to cirrhosis and stellate cells (Figure 2).

**Figure 3:**
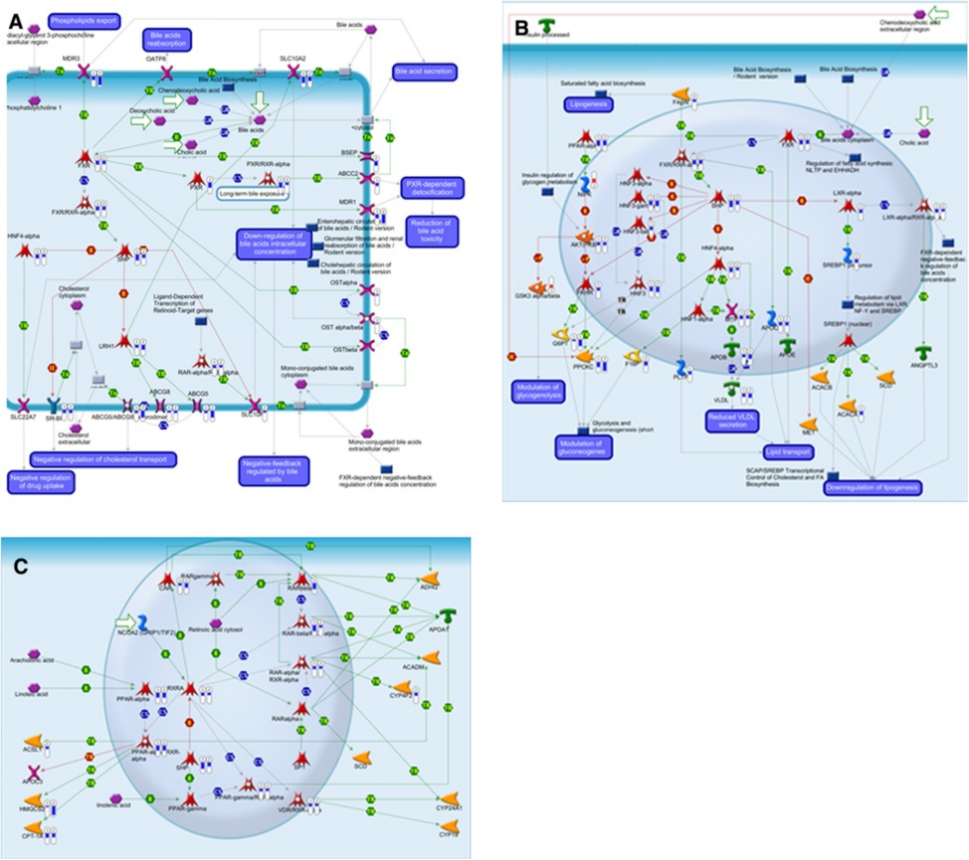
**Effects of cyclosporin A and chlorpromazine on the expression of genes involved in Fxr and Rxr-regulated pathways related to metabolism and/or transport of bile acids and lipids.** Cyclosporin A (CsA) and chlorpromazine (CPZ) significantly affected *Fxr-regulated cholesterol and bile acids cellular transport* pathway **(A)**, *Bile acids regulation of glucose and lipid metabolism via Fxr***(B)**, and *Rxr-dependent regulation of lipid metabolism via Ppar, Rar and Vdr* pathway **(C)**. Blue and red bars indicate down-and up-regulation respectively of significantly affected genes. 1 and 2 indicate liver slices treated with CsA and CPZ respectively. Each bar represents average fold change of gene expression (treatment vs. control) in liver slices from five mice. ECM: extracellular matrix. For an explanation of the MetaCore symbols is referred to http://pathwaymaps.com/pdf/MC_legend.pdf.

In order to get insight into the molecular mechanisms of action of CsA and CPZ, the significantly altered genes identified by GSEA (1192 and 811 genes for CsA and CPZ respectively) were uploaded into MetaCore to identify significantly altered pathways. In total, we identified 204 and 125 significantly altered pathways for CsA and CPZ, respectively (p < 0.005) (Additional file 6: Table S3). For a general overview of the processes affected by the drugs, the identified pathways were grouped into broader functional categories using a MetaCore pathway classification output (Table 1, Additional file 6: Table S3). The analysis based on the functional categories showed that there was a substantial overlap between pathways affected by the two drugs (Table 1). Both drugs affected pathways related to immune function (immune response, chemotaxis), energy metabolism (pathogenesis of obesity, regulation of metabolism), and other biological processes (development, apoptosis and survival, signal transduction, cell adhesion, G-protein signalling, protein folding and processing). CsA affected additional functional categories, such as cell cycle and DNA damage (Table 1). In a further analysis, we focused on those pathways that according to MetaCore pathway analysis, were found to be significantly altered either by both drugs or specifically by one drug (p < 0.005). Both CsA and CPZ altered *Fxr-regulated cholesterol and bile acids cellular transport* (p = 6.8E-07 and p = 7.5E-08 respectively), Figure 3A, Additional file 6: Table S3. In this pathway both CsA and CPZ significantly down-regulated Fxr as well as its target genes involved in BA transport such as solute carrier family 10 member 2 (Slc10a2) and multi-drug resistance 1/ ATP-binding cassette, sub-family B, member 1 (Mdr1/Abcb1), Figure 3A. ATP-binding cassette, sub-family C, member 2/ multidrug resistance-associated protein 2 (Abcc2/ Mrp2), were down-regulated only by CsA, while Bsep/ ATP-binding cassette, sub-family B, member 11(Abcb11) and solute carrier family 10 (sodium/bile acid cotransporter family), member 1/ Na + −taurocholate cotransporting polypeptide (Slc10a1/Ntcp) were down-regulated only by CPZ (Figure 3A). Moreover, both CsA and CPZ down-regulated genes coding for cholesterol transporters such as Abcg5/Abcg8 and Scavenger receptor class B member 1 (Sr-Bi) as well as genes encoding for phospholipids transporter- multidrug resistance 3/ ATP-binding cassette, sub-family B (Mdr/Tap), member 4 (Mdr3/ Abcb4/Mdr2), (Figure 3A). Another process significantly affected by both CsA and CPZ is *Bile acids regulation of glucose and lipid metabolism via Fxr* (p = 7.2E-14 and p = 1.1E-09 respectively), (Figure 3B). Both, CsA and CPZ led to down-regulation of several genes in this pathway, e.g. Fxr (Nr1h4), retinoid X receptor α (Rxrα), small heterodimer partner (Shp), hepatocyte nuclear factor 4 alpha (Hnf4α), apolipoprotein B (Apob), very low-density lipoprotein (VLDL/Apoc2), liver X receptor α (Lxr α). Furthermore, both CsA and CPZ significantly down-regulated another pathway involved in lipid homeostasis: *Rxr-dependent regulation of lipid metabolism via Ppar, Rar and Vdr* (p = 5.8E-05 and p = 2.9E-04 respectively), (Figure 3C, Additional file 6: Table S3).

**Table 1:** Overview of pathway functional categories affected by CsA and CPZ

**Functional category**	**Nr of pathways changed by CsA**	**Nr of pathways changed by CPZ**
Immune response	58	27
Development	53	41
Pathogenesis of obesity	23	16
Apoptosis and survival	18	6
Signal transduction	16	12
Regulation of metabolism	10	8
Cell cycle	5	0
Cell adhesion	4	8
G-protein signaling	4	2
Protein folding and processing	4	2
Chemotaxis	3	1
Cytoskeleton remodeling	3	2
DNA damage	2	0
Reproduction	1	0

Pathways identified in MetaCore were grouped into functional categories according to the MetaCore classification tool (first column). The number of affected pathways within the functional categories is presented in separate columns for cyclosporin A (CsA) and chlorpromazine (CPZ). MetaCore analysis revealed a total of 204 (CsA) and 125 (CPZ) significantly altered pathways (p < 0.005). All identified pathways are presented in Additional file 6: Table S3.

**Figure 4:**
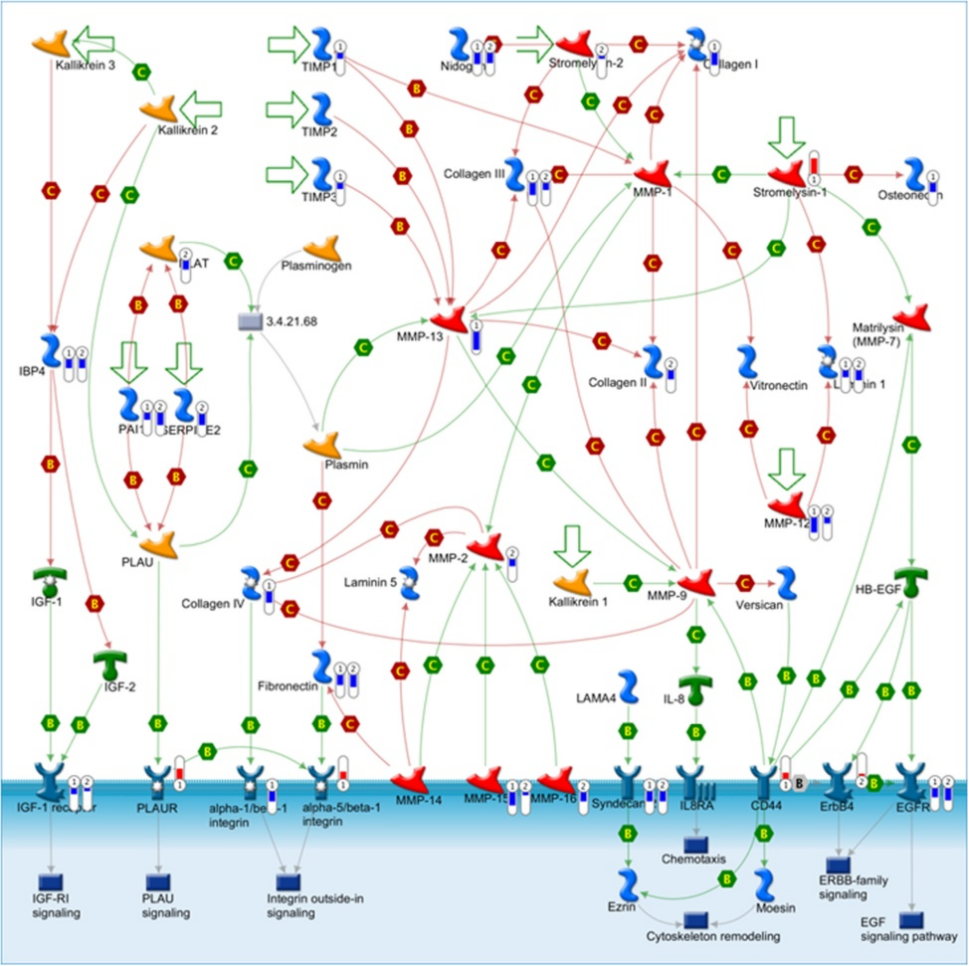
**Effects of cholestasis on the expression of genes involved in the Cell adhesion_ECM remodelling pathway and in mouse PCLS.** Both cyclosporin A (CsA) and chlorpromazine (CPZ) significantly affected the *Cell adhesion_ECM remodelling pathway* (p < 0.005)*.* Explanation of the bars is given in the legend of Figure 3.

In addition, CsA and CPZ significantly down-regulated several genes of *Cell adhesion_ECM remodelling pathway* (p = 1.3E-07 and p = 4.1E-09 respectively), (Figure 4, Additional file 6: Table S3). Several metalloproteinases were down-regulated by both drugs (Mmp-12, 15, 16), as well as other genes involved in ECM remodelling (Syndecan-2, Egfr).

One of the most significantly affected pathways by CsA was the *Endoplasmic reticulum stress response pathway* (p =1.8E-13), (Apoptosis and survival), Additional file 6: Table S3, Additional file 7: Figure S4. Several genes present in this pathway were up-regulated, such as NF-κB p50/p65, Grp78, Ire-1, and Traf2. Additionally, CsA affected several pathways related to NF-*κ*B signalling and inflammation, such as *NF-κB signaling* (p = 4.2E-11), *Il-1 signaling pathway* (p = 7.8E-11), and *Hsp60 and Hsp70 Tlr signaling pathway* (p = 1.7E-10), (Additional file 6: Table S3, Additional file 8: Figure S8A-C). In general we observed up-regulation of different NF-κB partners (NF-κB p65, p100, p52) and pro-inflammatory cytokines exampled by Tnfα or Il-12.

### Identification of biomarkers

**Figure 5:**
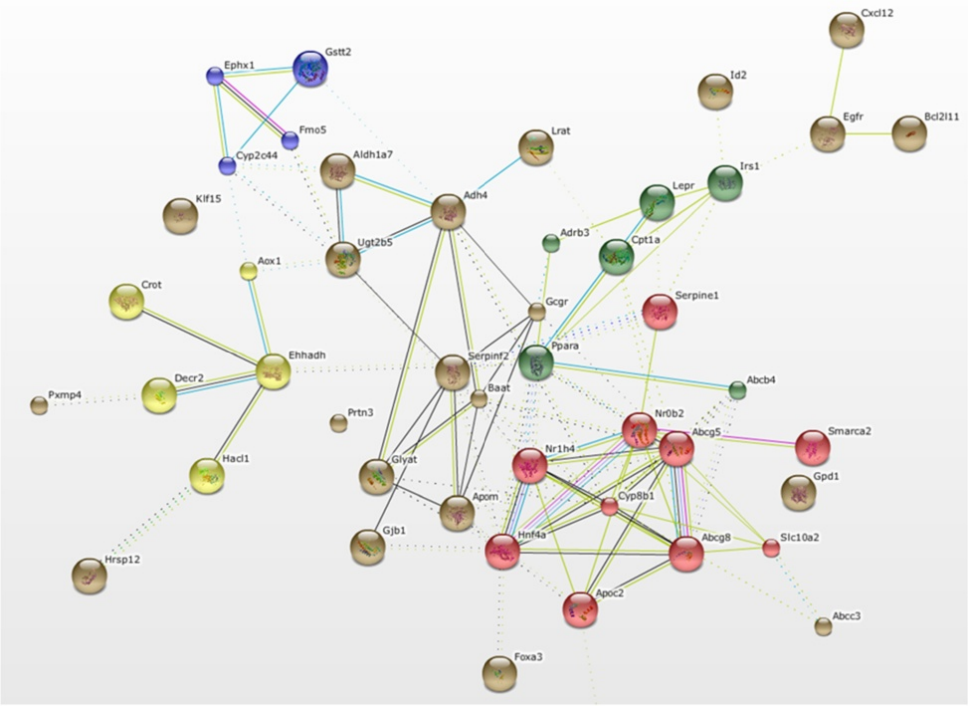
**Functional clustering of biomarkers.** In total 73 genes were identified as candidate biomarkers for drug-induced cholestasis. Clustering of biomarkers was performed in STRING using the functional clustering option. The analysis identified 5 functional clusters; yellow represents β-oxidation, blue represents biotransformation, brown represents bile acids and drugs conjugation, green represents lipid metabolism, and red regulation of bile acids metabolism via Fxr (Nr1h4). A total of 46 out of 73 genes formed connected nodes. Disconnected genes (27genes) were removed from the figure.

**Figure 6:**
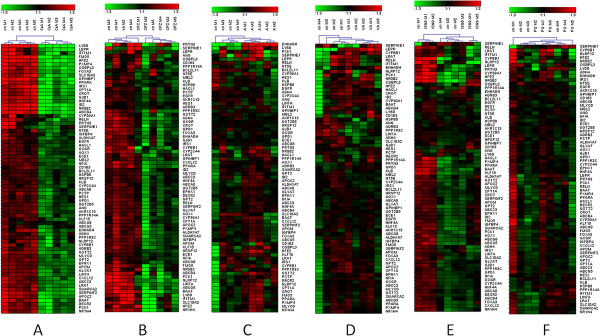
**Identification of potential biomarkers for cholestasis in PCLS.** PCLS were exposed for 24 hours to model toxicants for cholestasis (cyclosporin A (CsA)-**A**, chlorpromazine (CPZ)-**B**), steatosis (amiodarone (A)-**C**, valproic acid (VA)-**D**, necrosis, isoniazid (ISND-**E**), paraquat (PQ)-**F**, and controls (ctr)). GSEA led to the identification of 73 genes for which the mRNA expression was down-regulated by both CsA and CPZ. mRNA expression values for the selected biomarkers are derived from DNA-microarrays and results are presented as heat maps of log2, median centered gene expression values. Red and green indicate expression higher or lower respectively than the average expression of all samples within the same heat map. Please note Fxr is also depicted as Nr1h4.

In order to identify biomarkers that could be used for identification of drugs with cholestatic properties, genes were selected that, according to GSEA, significantly contributed to the enrichment of the analysed gene sets (p < 0.05, FDR < 0.25) in both CsA and CPZ treatments. In total 305 common candidate biomarkers were identified and further selection using FC ≥1.5 as criterion resulted in 73 genes. These genes could be categorized in five functional clusters: β-oxidation, biotransformation, BA metabolism, BA conjugation, and lipid metabolism (Figure 5). Hierarchical clustering of these genes, led to a very good separation between control and slices treated with CsA (Figure 6A). CPZ treated samples separated very well except for one control and one treated sample, which were mis-classified (Figure 6B). Furthermore, we analysed the expression levels of these 73 genes in liver slices exposed to two other classes of hepatotoxicants, i.e. steatogenic drugs (valproic acid and amiodarone) and necrotic drugs (paraquat and isoniazid). In contrast to the slices exposed to CsA and CPZ, hierarchical clustering of array data (of the 73 selected genes) from slices exposed to steatogenic (Figure 6C-D) and necrotic compounds (Figure 6E-F) did not show a clear separation between treatment and control.

### Microarray data verification (qPCR)

For verification of the array data, 4 genes out of the 73 potential biomarkers were subjected to qPCR. These genes were Baat, Abcg5, Abcg8, and Klf15. The major criterion for the selection of these genes was related to their function in Fxr signalling. Three of these four genes are related to BA metabolism and are targets of Fxr/Nr1h4 [2]. Moreover, mutations in Baat, Abcg5 or Abcg8 have been shown to be involved in the development of cholestasis in humans [2,8]. Although Klf15 is not a Fxr target, it is known to be involved in gluconeogenesis [27], which is regulated by Fxr [28,29]. More detailed information about the functions of the selected genes is presented in Additional file 9: Table S4. The qPCR experiments were performed on the same RNAs as used for the DNA microarray hybridisations. Statistical analysis of the qPCR data showed that CsA and CPZ significantly affected 3 out of the 4 candidate biomarkers (Table 2A-B). In slices exposed to necrotic and steatogenic drugs the expression of at most one out of the four genes was significantly affected (Table 2C-F).

**Table 2:** Validation of potential biomarkers for cholestasis in PCLS

**A**							**B**						
Drug	CsA	CsA	CsA	CsA	CsA	MW test	Drug	CPZ	CPZ	CPZ	CPZ	CPZ	MW test
Gene	FC1	FC2	FC3	FC4	FC5		Gene	FC1	FC2	FC3	FC4	FC5	
ABCG5	−3.5	−1.8	−1.0	−1.3	−1.2	NS	ABCG5	−4.2	−2.9	−6.7	−3.8	1.0	*
ABCG8	−4.9	−3.9	−1.2	−2.0	−1.2	*	ABCG8	−2.9	−2.1	−5.4	−4.5	−1.1	*
BAAT	−5.2	−4.2	−1.6	−2.2	−1.7	*	BAAT	−2.3	−3.3	−8.5	−6.4	1.4	*
KLF15	−1.2	−1.7	−2.0	−1.4	−1.3	*	KLF15	−2.0	−1.8	−3.2	−1.8	1.6	*
**C**							**D**						
Drug	A	A	A	A	A	MW test	Drug	VA	VA	VA	VA	VA	MW test
Gene	FC1	FC2	FC3	FC4	FC5		Gene	FC1	FC2	FC3	FC4	FC5	
ABCG5	1.2	−1.6	1.2	1.1	−1.3	NS	ABCG5	2.0	−1.7	−1.1	1.4	1.6	NS
ABCG8	2.0	−1.6	1.1	1.1	−1.1	NS	ABCG8	2.2	−1.5	−1.3	1.4	1.9	NS
BAAT	1.8	−2.2	1.1	−1.0	−1.7	NS	BAAT	1.3	1.0	−2.0	1.4	2.5	NS
KLF15	2.6	−1.8	−1.5	−1.4	−1.3	NS	KLF15	1.0	−1.9	−1.7	−1.1	1.1	NS
**E**							**F**						
Drug	ISND	ISND	ISND	ISND	ISND	MW test	Drug	PQ	PQ	PQ	PQ	PQ	MW test
Gene	FC1	FC2	FC3	FC4	FC5		Gene	FC1	FC2	FC3	FC4	FC5	
ABCG5	2.0	3.0	1.1	2.0	2.0	*	ABCG5	1.1	1.1	1.7	−1.3	−1.6	NS
ABCG8	2.1	2.8	1.0	2.3	1.9	NS	ABCG8	1.0	−1.2	1.8	−1.3	−1.4	NS
BAAT	1.8	1.7	1.1	−1.3	1.3	NS	BAAT	−1.2	−1.0	1.7	1.1	−1.4	NS
KLF15	1.2	2.4	1.0	1.1	1.2	NS	KLF15	1.3	−1.0	1.3	1.1	−1.2	NS

PCLS were exposed for 24 hours to model toxicants for cholestasis (cyclosporin A (CsA)-A, chlorpromazine (CPZ)-B), necrosis (isoniazid (ISND)-C, paraquat (PQ)-D), and steatosis (amiodarone (A)-E, valproic acid (VA)-F). Q-PCR was used to measure mRNA expression relative to actin β of four potential biomarkers: Abcg5, Abcg8, Baat, and Klf15. FC stands for fold change (treatment vs. control). Q-PCR was performed for liver slices obtained from five biological replicates. Asterisk (*) indicates significantly different gene expression (P < 0.05) from controls according to Mann–Whitney-test (MW). NS stands for not significant.

### Biochemical and histological analysis

**Figure 7:**
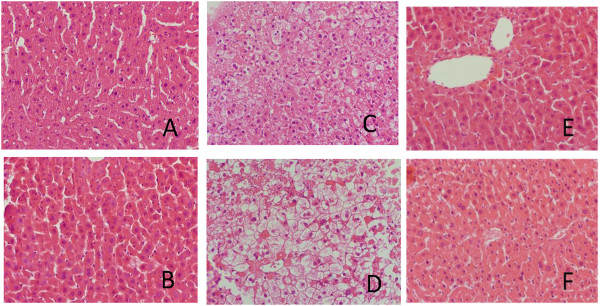
**Histological analysis of liver slices treated with CsA and CPZ.** PCLS were cultured for 24 and 48 hours in the presence of DMSO (control) (**A** and **B**), 40 μM CsA (**C** and **D**) or 20 μM CPZ (**E** and **F**). Histology of PCLS treated with CsA revealed ballooning of hepatocytes at the outer parts of slices after 24 and 48 hours (**C** and **D** respectively). Histology of CPZ treated slices cultured for 24 and 48 hours (**E** and **F** respectively) revealed a slight increase in number of cells containing pycnotic nuclei compared to control slices cultured equally long (**A** and **B**).

**Figure 8:**
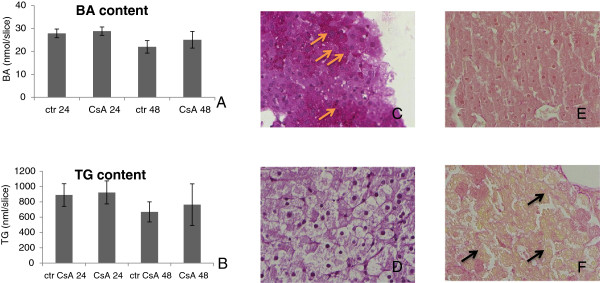
**Biochemical analysis and specific histological staining of liver slices treated with CsA.** Bile acid (BA) **(A)** and triglyceride (TG) **(B)** levels in PCLS cultured for 24 or 48 hours in the presence of either DMSO or 40 μM cyclosporin A (CsA) were not affected by any of the conditions, X- axis represents the tested conditions and Y-axis represents content of BA **(A)** or TG **(B)** in nmol per slice. Glycogen was assessed by PAS staining in PCLS cultured for 24 h in the presence of DMSO **(C)** or 40 μM CsA **(D)** demonstrating the absence of glycogen in ballooning hepatocytes. Arrows in **(C)** indicate the presence of glycogen in controls. Fouchet staining in PCLS cultured for 24 h in the presence of DMSO **(E)** or 40 μM CsA **(F)** demonstrating accumulation of several vacuoles in CsA treated slices (see arrows in **F**). All pictures were taken using 100-fold magnification.

Slices cultured for 24 and 48 hours with the reference compounds and controls were subjected to histological analysis. Upon incubation for 24 hours, some hepatocytes localized in the outer part of slices treated with CsA had developed a characteristic ballooned phenotype, which was even more evident after 48 hours and was absent in control slices. These hepatocytes were enlarged compared to control and had their nucleus in the centre (Figure 7). Based on the histological examination as well as transcriptome data analysis, showing alternations in lipid and BA metabolism, it could be envisaged that the enlargement of cells treated with CsA is due to TG or BA accumulation. Therefore, biochemical assays to quantify TG and BA levels were employed. However, compared to control, no increase of BA or TG in CsA treated slices was observed (Figure 8A and B). Additionally, we performed Periodic acid-Schiff (PAS) staining to detect glycogen, which theoretically could also accumulate in hepatocytes and lead to their enlargement. As expected, control slices showed substantial number of cells containing glycogen (Figure 8C). However, in the enlarged cells of the CsA treated slices no glycogen could be detected (Figure 8D). Finally, Fouchet staining was performed to examine whether CsA treatment resulted in the accumulation of bile, a process that is often accompanying cholestasis. Although, we did not detect bile accumulation in slices, Fouchet staining unexpectedly showed the presence of several vacuoles, which were absent in control slices (Figure 8E and F).

### Comparative data analysis: relevance for human pathology

**Figure 9:**
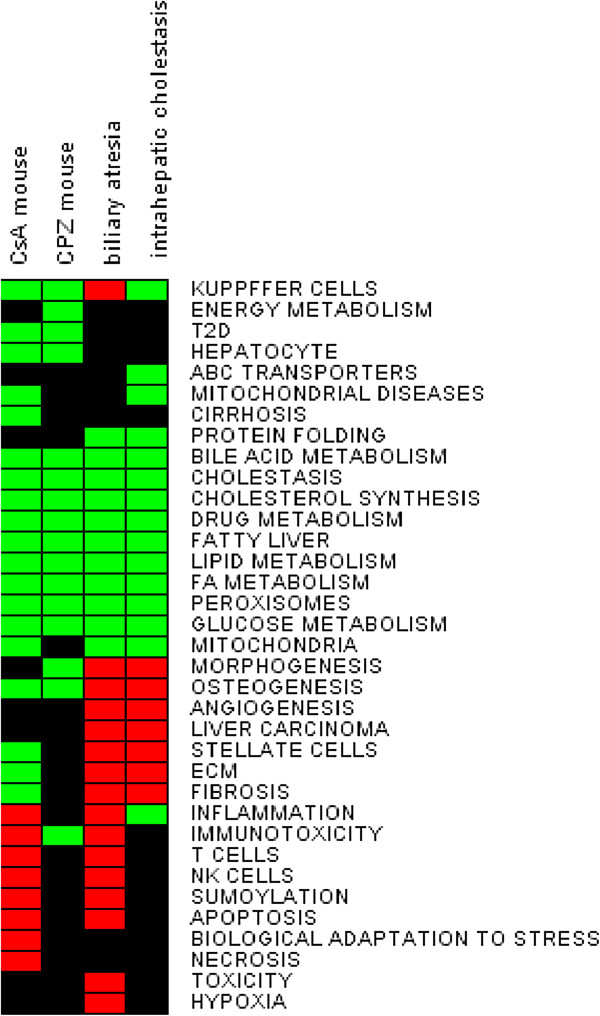
**Effects of cholestasis on gene expression in human liver biopsies.** A publically available transcriptomics data set (GSE46960) of liver biopsies obtained from 64 infants with biliary atresia, 14 infants with intrahepatic cholestasis of other origin than biliary atresia, and 7 age-matched controls were subjected to GSEA. The biological processes in the heat map correspond to gene sets significantly affected according to GSEA analysis (p < 0.05, FDR < 0.25). Gene sets were obtained using the ANNI text mining tool. Processes that were up-regulated are represented by red color, the down-regulated processes are depicted in green, and processes that were unchanged are depicted in black.

In order to verify whether the results on drug induced cholestasis in mouse liver slices would also be observed in patients suffering from cholestasis, publically available transcriptomics data were analysed. The data were generated using liver biopsies obtained from children suffering from biliary atresia, intrahepatic cholestasis of other origin than biliary atresia, and age-matched controls. GSEA revealed that patients suffering from both biliary atresia and intrahepatic cholestasis had significantly down-regulated gene sets related to different aspects of energy metabolism including bile acid metabolism, FA metabolism or peroxisomes. These changes were in line with drug-induced cholestasis in mouse PCLS (Figure 9). However, gene sets related to ECM, angiogenesis, and fibrosis were up-regulated in the livers of cholestatic patients but the same processes were either not affected or altered in the opposite direction by the tested drugs in mouse liver slices (Figure 9). MetaCore pathway analysis showed that FXR signalling such as *Bile acids regulation of glucose and lipid metabolism via FXR* (Additional file 10: Figure S6A) and *FXR-dependent negative-feedback regulation of bile acids concentration* (Additional file 10: Figure S6B) were significantly down-regulated in cholestatic patients. These latter two pathways were also found to be down-regulated in the mouse slices experiment (Figure 3). Furthermore, not only GSEA but also MetaCore analysis showed that the pathway *ECM remodeling* was up-regulated (Additional file 11: Figure S7). This pathway was down- regulated by CsA -and CPZ-treatment in mouse PCLS (Figure 4).

## Discussion

In the present study, we aimed to assess whether mouse PCLS can be used as an *ex vivo* model to study processes related to the development of drug-induced cholestasis. CsA and CPZ were used as reference compounds known to induce cholestasis *in vivo* in humans and rodents. In depth transcriptome analysis combined with biochemical and histological evaluation were performed to identify early molecular events and biomarkers that can be used to screen drugs for cholestatic properties.

### Exposure of PCLS to CsA and CPZ

The effects of CsA and CPZ were analysed upon treatment of PCLS with 40 μM CsA and 20 μM CPZ. These concentrations were relatively high compared to plasma levels of patients chronically treated with the same drugs, i.e. 0.04-0.1 μM for CsA [26] and 0.02-0.3 μM for CPZ [30], but similar concentrations were used in other *in vitro* studies [30,31] and the applied concentrations did not cause a decrease in slices viability assessed ATP and LDH assays.

However, GSEA analysis on the transcriptome data revealed that CsA induced a higher number of processes indicative for cytotoxicity such as apoptosis, necrosis, or inflammation than CPZ (Figure 2). This could indicate that the applied concentration of CsA was toxic, although according to the viability tests (ATP and LDH assays) doses even higher than 40uM did not cause a significant decrease in viability (Additional file 1: Figure S1). These results suggest therefore, that ATP as well as LDH measurements may not always be reliable to predict cytotoxic concentration of drugs. In addition, due to known interspecies differences for toxic doses of drugs, application of drug concentrations identical to *in vitro* studies using different species, seems to be inappropriate as well [32,33]. On the other hand, we cannot exclude that the cytotoxic processes are due to pharmacological actions of CsA, because other in vitro studies demonstrated that also lower CsA concentrations induce toxicity-related processes such as ER stress, apoptosis, and cystic fibrosis [26].

Subsequent detailed pathway analysis revealed that both CsA and CPZ significantly altered pathways (p < 0.005) governed by Fxr including *Fxr regulated cholesterol and BAs cellular transport* (Figure 3A, Additional file 6: Table S3) and *BA regulation of glucose and lipid metabolism via Fxr* (Figure 3B, Additional file 6: Table S3). Both drugs down-regulated several transporters involved in bile production and BA secretion: Mdr1, Abcg5/Abcg8, as well as Mdr3 (Mdr2). This is in agreement with a previous rat PCLS study, which showed that CsA reduced Mdr1 activity [30]. Furthermore, we observed that CsA down-regulated another BA transporter, Mrp2 (Abcc2). These data are in line with observations made in rats showing that the same transporters were down-regulated by CsA in the liver at the protein level [34]. With regard to CPZ we detected down-regulation of Fxr targets such as Bsep, Mdr3, Ntcp (Slc10a1), and Cyp8b1. These findings are in agreement with a recent study showing a down-regulation of the same genes in CPZ-treated HepaRG cells [35]. The importance of Fxr signalling in the maintenance of BA homeostasis *in vivo* is unequivocal and it was demonstrated that Fxr knockout mice have disturbed BA homeostasis [36]. Moreover, we observed that both CsA and CPZ down-regulated several genes related to lipid metabolism (e.g. Hnf4α, Apob, Apoc, Hnf3, or Pparα) [2] in *BA regulation of glucose and lipid metabolism via Fxr pathway* (Figure 3B, Additional file 6: Table S3). Consistent with these observations, it was reported that CsA treatment led to an increase in accumulation of lipid droplets in rat primary hepatocytes after 24 hours [37] and CsA therapy has been implicated in the development of fatty liver [38]. Next to that, CPZ has been shown to inhibit β-oxidation *in vivo*[39] and to evoke an increase in microsomal phospholipidosis in rats [40]. However, based on biochemical measurement of TG in our study, we concluded that no lipids accumulated in CsA or CPZ treated slices. Based on the outcome of the present work, we propose that CsA and CPZ down-regulate Fxr signalling resulting in perturbation of BA, glucose, and lipid metabolism, which eventually leads to the development of both cholestasis and metabolic disorders. This is in line with the known functions of Fxr related to the regulation of both BA and energy homeostasis [10,36] and common side effects of CsA and CPZ treatments, i.e.cholestasis, fatty liver, and type 2 diabetes [38,40]. Additionally, we detected that both CsA and CPZ down-regulated other NRs involved in detoxification of BA and lipid metabolism, such as Car (Nr1i3), Vdr and other genes related to lipid metabolism such as Cpt1a and Hmgcs2 [9], (depicted in *Rxr-dependent regulation of lipid metabolism via Ppar, Rar and Vdr* pathway, Figure 3C, Additional file 6: Table S3). Down-regulation of these genes possibly adds to the cholestatic and lipogenic properties of the analysed drugs.

Furthermore, for both CsA and CPZ, significant alternations in ECM remodelling pathway were found (Figure 4). It was suggested before, that changes related to ECM remodelling could lead to perturbations in intracellular signalling affecting Fxr related pathways and thereby leading to liver injuries [41].

Transcriptome data analysis also revealed processes and pathways that were specifically affected by either CsA or CPZ. CsA treatment up-regulated pathways and genes related to endoplasmic reticulum (ER) stress (Additional file 7: Figure S4), NF-κB signalling, and inflammation with upregulated cytokines such as Tnfα, Crp, and Il-1β (Additional file 8: Figure S5A-C). These findings are in line with previous reports describing ER stress and inflammation as the primary events in CsA induced toxicity [42,43]. ER stress is known to activate NF-κB, which is a key regulator of diverse immune responses [44]. Moreover, we found that CsA up-regulated gene sets related to T cells. This contrasts to findings that CsA inhibits T-cell receptor signalling and induces Tgfβ, which both prevent the production of cytokines and block the immune system [42]. Inasmuch as CsA caused up-regulation of gene sets related to functions of T cells, the immunosuppressive effects of CsA in our model, seem to be different compared to CsA effects *in vivo*. A possible cause could be the relatively short time of this study or a lack of blood immune cells which interactions with the liver immune cells are necessary to fully develop the immune response [45]. To the best of our knowledge, there is only one report describing presence and actions of T cells in human liver slices [46]. Therefore, the exact biological meaning of the CsA effect on gene expression specific to T-cells in mouse PCLS remains to be elucidated in the future. Additionally, CsA down-regulated the expression of several genes encoding heat shock proteins (HSP) (e.g. Hspb6, Hsph1, Hspe1) as well as Sec proteins (Sec61a1, Sec14l2, Sec14l4) (shown in Additional file 12). HSP proteins and Sec proteins are involved in folding/ unfolding of proteins and protein translocation, respectively [47,48]. It is known that ER stress induced by CsA negatively affects the function of the HSP and Sec proteins that eventually lead to perturbations in protein and bile secretion [49,50]. Also the vacuolisation in CsA treated slices, as shown in the present study by Fouchet staining, could be a consequence of this disturbance in protein folding and translocation/ secretion [26,49,51].

The up-regulation of ER stress, NF-κB signalling and expression of pro-inflammatory cytokines (e.g. Tnfα, Crp, Il1β) by CsA could be the cause of the observed down-regulation of Fxr and its gene targets as well as other nuclear receptors. Previously it has been shown that the NF-κB-mediated acute phase response induced by lipopolysaccharide (LPS) is associated with a decrease of Fxr expression in mouse liver [52]. Furthermore, TNFα and IL1β treatment of Hep3B human hepatoma cells and mice resulted in decreased expression of RXRα, PPARα, PPARγ, LXRα, as well as their co-activators PGC1α and PGC-1β, which may contribute to the cytokine induced modulations in hepatic energy homeostasis [53].

Although CPZ did not induce oxidative stress based on transcritpome data, it is likely that it is also a trigger to down-regulate Fxr signaling at early time points that we did not test. Consistent with this notion, it was shown that CPZ induces oxidative stress in HepaRG cells already after 30 min resulting in down-regulation of FXR-target genes after 24 hours [35].

Moreover, we observed that CPZ and CsA down-regulated gene sets related to Kupffer cells (Figure 2) pointing out towards known immunosuppressive actions of CPZ and CsA [54]. Analysis of the microarray data to identify potential biomarkers for cholestasis showed a substantial number of genes separating the two cholestatic compounds from non-cholestatic hepatotoxicants (Figure 6A-F). Functional clustering of these potential biomarkers identified processes such as β-oxidation, biotransformation, BA metabolism and conjugation, and lipid metabolism. To verify the gene expression data obtained with microarrays, q-PCR was performed on genes involved in processes governed by Fxr i.e. BA homeostasis and gluconeogenesis [2,27,29]. The expression of the selected biomarkers as assessed by q-PCR was similar to that of the microarray analysis (Tables 2 A-F). To further validate the potential of these genes (Figure 6) to screen for cholestasis additional studies are required using more reference compounds and models.

### Biochemical and histological analysis

Based on the transcriptome data, it was expected that TG or BA levels would be altered in PCLS upon the treatment with CsA and CPZ. However, this could not be confirmed by biochemical and histological assays. Fouchet staining, reacting with bilirubin is not suitable to identify bile accumulation *ex vivo* probably due to lack of bilirubin derived from broken heme that *in vivo* reaches the liver via the blood. On the other hand, H&E staining demonstrated that CsA, but not CPZ, induced ballooning of hepatocytes. This phenotype is generally regarded as a form of apoptosis in the context of inflamed fatty liver (steatohepatitis) caused by obesity, alcohol or other toxic compounds [55,56]. This phenotype could be associated with the up-regulation of gene sets related to apoptosis and inflammation observed in CsA treated slices.

It has to be underlined that the mouse PCLS experiments were performed without supplementing the media with BA. On the one hand this might explain the absence of BA accumulation in the slices upon treatment with CsA and CPZ. On the other hand, by leaving out BA from the culture medium, we have identified early events involved in the development of CsA- and CPZ-induced cholestasis rather than indirect effects associated with a late cholestatic phenotype, such as toxic BA accumulation.

### Relevance of the mouse PCLS findings for human

Due to the lack of transcriptomic data of cholestatic livers from patients treated with CsA and CPZ, we used publically available data of livers of patients suffering from cholestasis due to biliary atresia and other causes. The comparison of the gene expression profiles of drug-induced cholestasis in mouse PCLS and livers of cholestatic patients pointed to several processes that were similarly affected. These processes are associated with energy metabolism and include lipid, BA metabolism, and glucose metabolism, among others (Figure 9, Additional file 10: Figure S6). The comparative analysis thereby indicates that mouse PCLS are a valid model to study mechanisms involved in the development of human cholestasis. The opposite direction in regulation of gene sets and pathways related to ECM, angiogenesis and fibrosis between livers of cholestatic patients and mouse PCLS (Figure 9, Additional file 11: Figure S7) could be explained by obvious differences related to e.g. chronic pathological conditions *in vivo* vs. short time experiments in PCLS. The same is true for up-regulation, only in patients, of other gene sets related to liver carcinoma, angiogenesis or fibrosis (Figure 9) that, in order to develop, require long-term processes as well as multi-organ and blood cells interactions [2,57]. Consistent with these notions, it was also reported that cholestasis and other chronic liver diseases are associated with the development of fibrosis caused by accumulation of ECM [58].

However, whether oxidative stress and subsequent down-regulation of NRs involved in the maintenance of BA and energy homeostasis are the common links between drug-related cholestasis in mouse (PCLS) and drug-unrelated cholestasis in humans, remains to be resolved in the future, when more appropriate human data will be available.

## Conclusion

In summary, our study demonstrates that mouse PCLS can be used as a tool to identify mechanisms of action of cholestatic compounds. Based on the transcriptome analysis it is proposed that CsA and CPZ affect pathways involved in general stress responses, such as ER stress (CsA), NF-κB-mediated responses (CsA), and ECM remodelling (CsA and CPZ) associated with down-regulation of Fxr signalling, a key process in BA and lipid homeostasis. Importantly, gene expression pattern in livers of cholestatic patients displayed several similarities to PCLS treated with model cholestatic compounds, indicating that some common mechanisms are involved in the development of cholestasis both in human pathology and mouse PCLS. Moreover, this work provides a set of genes that are potentially useful to screen compounds for Fxr-mediated cholestatic properties.

## Competing interests

The authors declare that they have no competing interests.

## Authors’ contributions

ESZ designed the study, performed experiments, analyzed data, wrote the manuscript, GS performed experiments, MG analyzed data, PH and AP analyzed data and edited the manuscript. All authors read and approved the final manuscript.

## Pre-publication history

The pre-publication history for this paper can be accessed here:

http://www.biomedcentral.com/1755-8794/6/39/prepub

## Supplementary Material

Additional file 1: Figure S1 A-F. Dose selection experiments for cholestatic drugs. Biochemical viability assays in liver slices after 24 hours exposure to cyclosporin A (CsA) and chlorpromazine (CPZ). Liver slices were incubated for 24 hours and exposed to different concentrations of cyclosporin A (CsA) (0–100 μM) or chlorpromazine CPZ (0-80uM) and compared to 0.1% DMSO control. Slices viability was assessed by protein content for CsA and CPZ (A and B respectively), ATP content for CsA and CPZ (C and D respectively) and LDH leakage for CsA and CPZ (E and F respectively). Each point is ± SD of two independent experiments (liver slices were isolated from livers of two mice, additionally for each measurement three technical replicates were used). Error bars represent ± SD. Click here for file

Additional file 2: Figure S2 A-C. Dose selection experiments for steatogenic drugs. Biochemical viability assays in liver slices after 24 hours exposure to amiodarone (A) and valproic acid (VA). Liver slices were incubated for 24 hours and exposed to different concentrations of A (0–100 μM) or VA (0–500 μM) and compared to corresponding controls. Slices viability was assessed by protein content, ATP content and LDH leakage. Each point is ± SD of five independent experiments (liver slices were isolated from livers of five mice, additionally for each measurement two technical replicates were used). Click here for file

Additional file 3: Figure S3 A-C. Dose selection experiments for necrotic drugs. Biochemical viability assays in liver slices after 24 hours exposure to isoniazyd (ISND) and paraquat (PQ). Liver slices were incubated for 24 hours and exposed to different concentrations of ISND (0–1000 μM) or PQ (0–10 μM) and compared to corresponding controls. Slices viability was assessed by protein content, ATP content, and LDH leakage. Each point is ± SD of five independent experiments (liver slices were isolated from livers of five mice, additionally for each measurement two technical replicates were used). Click here for file

Additional file 4: Table S1 ANNI gene sets. Click here for file

Additional file 5: Table S2 List of primers used for q-PCR. Click here for file

Additional file 6: Table S3 Total pathway analysis. Pathway analysis led to identification of 204 and 125 significantly altered pathways for CsA (A) and CPZ (B) (p < 0.005). Pathways were grouped into functional categories according to MetaCore pathways classification (first column). Each functional category contains pathway name and p value. Additionally number of significantly affected genes and total number of gene in pathways are given. Pathways that were discussed in the paper in details are in bold and underlined. Click here for file

Additional file 7: Figure S4 MetaCore pathway analysis in CsA treated slices: *Endoplasmic reticulum stress response pathway*. Cyclosporin A (CsA) significantly affected *Endoplasmic reticulum stress response pathway* (p < 0.005). Blue (down-regulation) and red (up-regulation) bars indicate significantly affected genes. The numbers 1–5 represent fold change (treatment vs. control) of gene expression of five independent experiments (i.e. liver slices isolated from 5 mice). Click here for file

Additional file 8: Figure S5 MetaCore pathway analysis in CsA treated slices: *Nf-κB signaling Il-1 signaling pathway*, and *Hsp60 and Hsp70 Tlr signaling* pathways. PCLS treated with CsA displayed significant (p < 0.005) up regulation of *NF-κB signaling* (A*, Il-1 signaling pathway* (B), and *Hsp60 and Hsp70 Tlr signaling* pathways (C). Blue (down-regulation) and red (up-regulation) bars indicate significantly affected genes. The numbers 1–5 represent fold change (treatment vs. control) of gene expression of five independent experiments (i.e. liver slices isolated from 5 mice). Click here for file

Additional file 9: Table S4 Functions of genes tested by q-PCR, source GeneCards http://www.genecards.org. Click here for file

Additional file 10: Figure S6 A-B. Effects of cholestasis on the expression of genes involved in Fxr-regulated pathways related to lipid and bile acids metabolism in human liver biopsies. Both biliary atresia and intrahepatic cholestasis significantly down-regulated *Bile acids regulation of glucose and lipid metabolism via Fxr* (A), and *Fxr-dependent negative-feedback regulation of bile acids concentration* (B). Blue and red bars indicate down-and up-regulation respectively of significantly affected genes. The bar numbers 1 and 2 indicate liver biopsies obtained from patients suffering from biliary atresia and intrahepatic cholestasis, respectively. Each bar represents average fold change of gene expression (disease vs. control) in liver biopsies. For an explanation of the MetaCore symbols is referred to http://pathwaymaps.com/pdf/MC_legend.pdf. Click here for file

Additional file 11: Figure S7 A. *Cell adhesion ECM remodelling pathway* is affected in liver biopsies of patients suffering from cholestasis. In two types of human cholestasis, biliary atresia (bar 1) and intrahepatic cholestasis (bar 2) both *Cell adhesion_ECM remodelling pathway* (p < 0.005) (A) was affected*.* Explanation of the bars is given in the legend of Additional file 10: Figure S6. Click here for file

Additional file 12 **Microarray data from PCLS exposed to cholestatic model compounds. **Microarray data were generated using liver slices obtained from livers obtained from 5 mice per one experimental condition. Abbreviations used: M1-5 stands for mouse 1–5, ctr stands for control, CsA CyclosporinA, CPZ chlorpromazine, 24 means 24 h incubation time. Data are normalized and presented as log2 values of intensities. Click here for file
