# Supplementary material for: Treatment of mouse liver slices with cholestatic hepatotoxicants results in down-regulation of Fxr and its target genes
Source: BMC Med Genomics. 2013 Oct 10;6:39. doi: 10.1186/1755-8794-6-39 (PMC3852711; doi:10.1186/1755-8794-6-39)
Supplement: Additional file 1: Figure S1 — A-F. Dose selection experiments for cholestatic drugs. Biochemical viability assays in liver slices after 24 hours exposure to cyclosporin A (CsA) and chlorpromazine (CPZ). Liver slices were incubated for 24 hours and exposed to different concentrations of cyclosporin A (CsA) (0–100 μM) or chlorpromazine CPZ (0-80uM) and compared to 0.1% DMSO control. Slices viability was assessed by protein content for CsA and CPZ (A and B respectively), ATP content for CsA and CPZ (C and D respectively) and LDH leakage for CsA and CPZ (E and F respectively). Each point is ± SD of two independent experiments (liver slices were isolated from livers of two mice, additionally for each measurement three technical replicates were used). Error bars represent ± SD. [file 1755-8794-6-39-S1.pptx]

## Slide 1
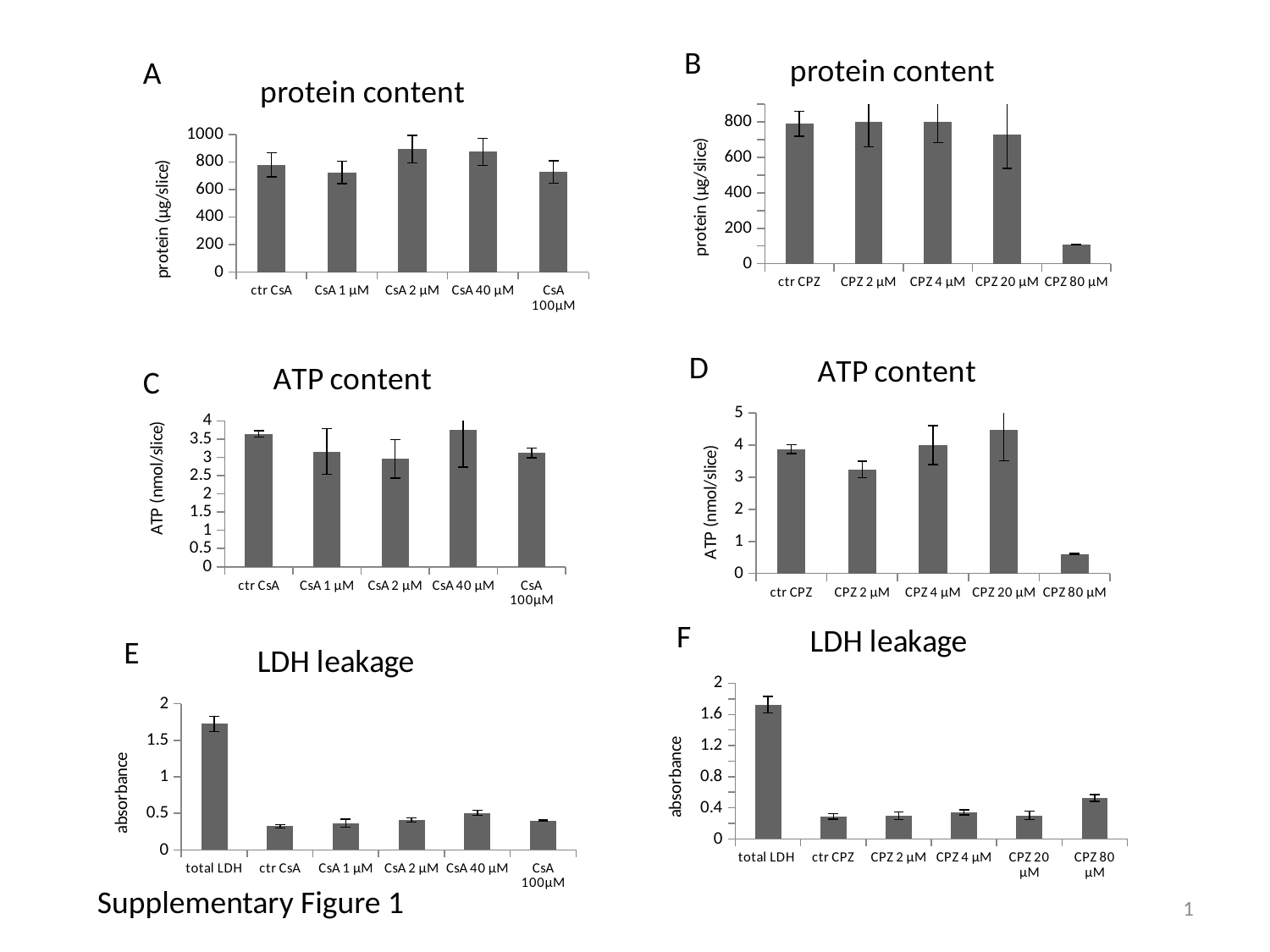

### Chart: protein content
| Category | |
|---|---|
| ctr CPZ | 789.3364571428571 |
| CPZ 2 µM | 800.7268571428572 |
| CPZ 4 µM | 801.3596571428573 |
| CPZ 20 µM | 730.4860571428571 |
| CPZ 80 µM | 109.07645714285715 |B
A
### Chart: protein content
| Category | |
|---|---|
| ctr CsA | 777.4059770666669 |
| CsA 1 µM | 724.5136000000001 |
| CsA 2 µM | 892.8384000000002 |
| CsA 40 µM | 873.6434666666667 |
| CsA 100µM | 725.9901333333335 |
### Chart: ATP content
| Category | |
|---|---|
| ctr CPZ | 3.877075 |
| CPZ 2 µM | 3.2428392857142856 |
| CPZ 4 µM | 4.002975 |
| CPZ 20 µM | 4.4667 |
| CPZ 80 µM | 0.6005491071428571 |D
### Chart: ATP content
| Category | |
|---|---|
| ctr CsA | 3.646588235294118 |
| CsA 1 µM | 3.1620000000000004 |
| CsA 2 µM | 2.9648571428571433 |
| CsA 40 µM | 3.7572631578947373 |
| CsA 100µM | 3.1203000000000003 |C
### Chart: LDH leakage
| Category | |
|---|---|
| total LDH | 1.725 |
| ctr CPZ | 0.291 |
| CPZ 2 µM | 0.29900000000000004 |
| CPZ 4 µM | 0.3425 |
| CPZ 20 µM | 0.30300000000000005 |
| CPZ 80 µM | 0.528 |F
E
### Chart: LDH leakage
| Category | |
|---|---|
| total LDH | 1.725 |
| ctr CsA | 0.324 |
| CsA 1 µM | 0.36550000000000005 |
| CsA 2 µM | 0.4105 |
| CsA 40 µM | 0.5075000000000001 |
| CsA 100µM | 0.4025 |Supplementary Figure 1
1
