# Supplementary material for: Treatment of mouse liver slices with cholestatic hepatotoxicants results in down-regulation of Fxr and its target genes
Source: BMC Med Genomics. 2013 Oct 10;6:39. doi: 10.1186/1755-8794-6-39 (PMC3852711; doi:10.1186/1755-8794-6-39)
Supplement: Additional file 2: Figure S2 — A-C. Dose selection experiments for steatogenic drugs. Biochemical viability assays in liver slices after 24 hours exposure to amiodarone (A) and valproic acid (VA). Liver slices were incubated for 24 hours and exposed to different concentrations of A (0–100 μM) or VA (0–500 μM) and compared to corresponding controls. Slices viability was assessed by protein content, ATP content and LDH leakage. Each point is ± SD of five independent experiments (liver slices were isolated from livers of five mice, additionally for each measurement two technical replicates were used). [file 1755-8794-6-39-S2.pptx]

## Slide 1
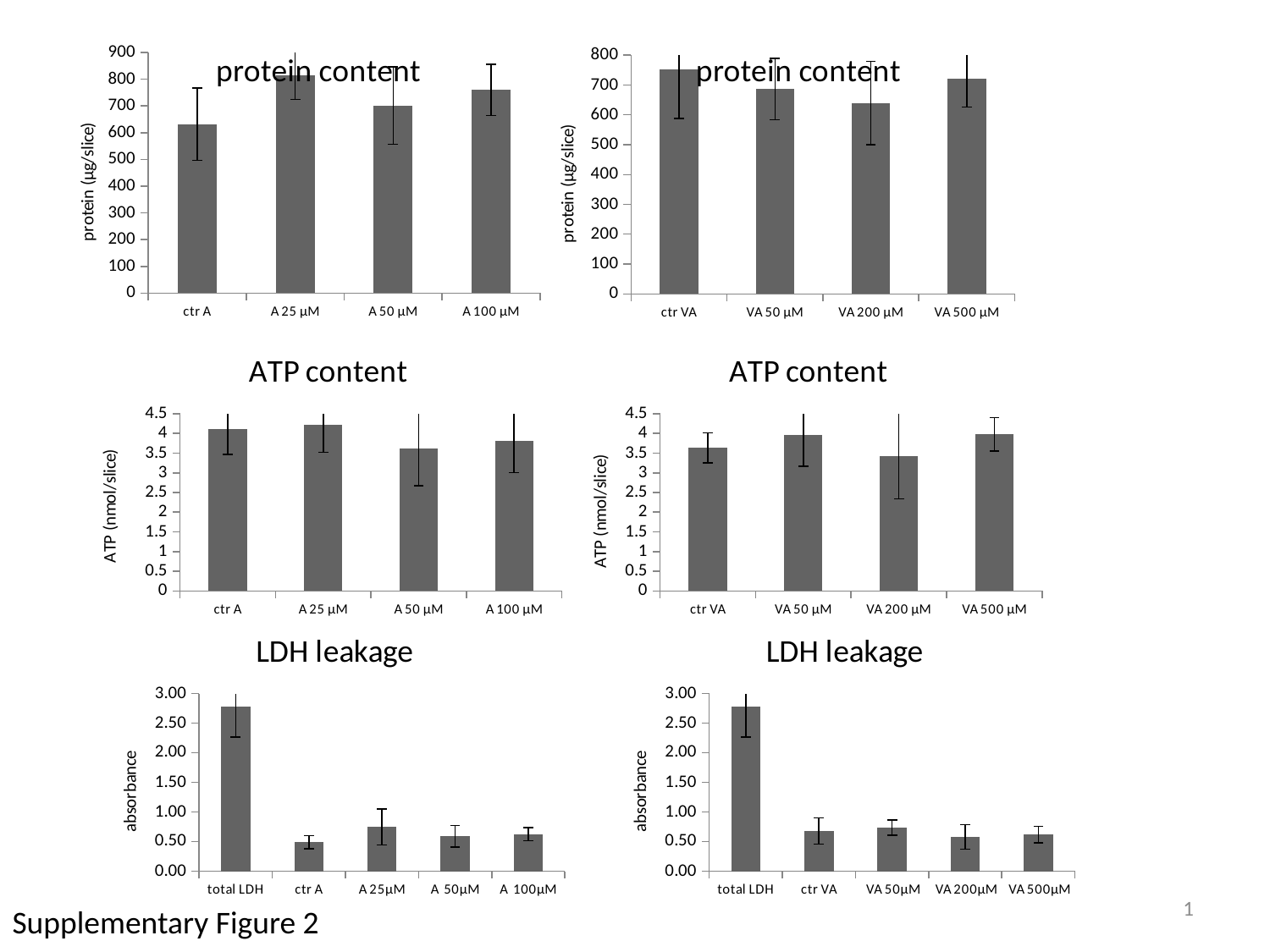

### Chart: protein content
| Category | |
|---|---|
| ctr A | 632.0321066666667 |
| A 25 µM | 816.0501599999998 |
| A 50 µM | 701.6283306666667 |
| A 100 µM | 759.9504000000001 |
### Chart: protein content
| Category | |
|---|---|
| ctr VA | 751.4947840000001 |
| VA 50 µM | 686.4515839999998 |
| VA 200 µM | 639.4036693333333 |
| VA 500 µM | 721.7917226666666 |
### Chart: ATP content
| Category | |
|---|---|
| ctr A | 4.119465 |
| A 25 µM | 4.21644 |
| A 50 µM | 3.6114000000000006 |
| A 100 µM | 3.8189400000000004 |
### Chart: ATP content
| Category | |
|---|---|
| ctr VA | 3.6297150000000005 |
| VA 50 µM | 3.953265 |
| VA 200 µM | 3.4212000000000002 |
| VA 500 µM | 3.98016 |
### Chart: LDH leakage
| Category | |
|---|---|
| total LDH | 2.7800000000000002 |
| ctr A | 0.4908 |
| A 25µM | 0.7458 |
| A 50µM | 0.5888666666666666 |
| A 100µM | 0.6256 |
### Chart: LDH leakage
| Category | |
|---|---|
| total LDH | 2.7800000000000002 |
| ctr VA | 0.6767333333333334 |
| VA 50µM | 0.7363999999999999 |
| VA 200µM | 0.5785333333333333 |
| VA 500µM | 0.6182000000000001 |1
Supplementary Figure 2
