# Supplementary material for: Treatment of mouse liver slices with cholestatic hepatotoxicants results in down-regulation of Fxr and its target genes
Source: BMC Med Genomics. 2013 Oct 10;6:39. doi: 10.1186/1755-8794-6-39 (PMC3852711; doi:10.1186/1755-8794-6-39)
Supplement: Additional file 3: Figure S3 — A-C. Dose selection experiments for necrotic drugs. Biochemical viability assays in liver slices after 24 hours exposure to isoniazyd (ISND) and paraquat (PQ). Liver slices were incubated for 24 hours and exposed to different concentrations of ISND (0–1000 μM) or PQ (0–10 μM) and compared to corresponding controls. Slices viability was assessed by protein content, ATP content, and LDH leakage. Each point is ± SD of five independent experiments (liver slices were isolated from livers of five mice, additionally for each measurement two technical replicates were used). [file 1755-8794-6-39-S3.pptx]

## Slide 1
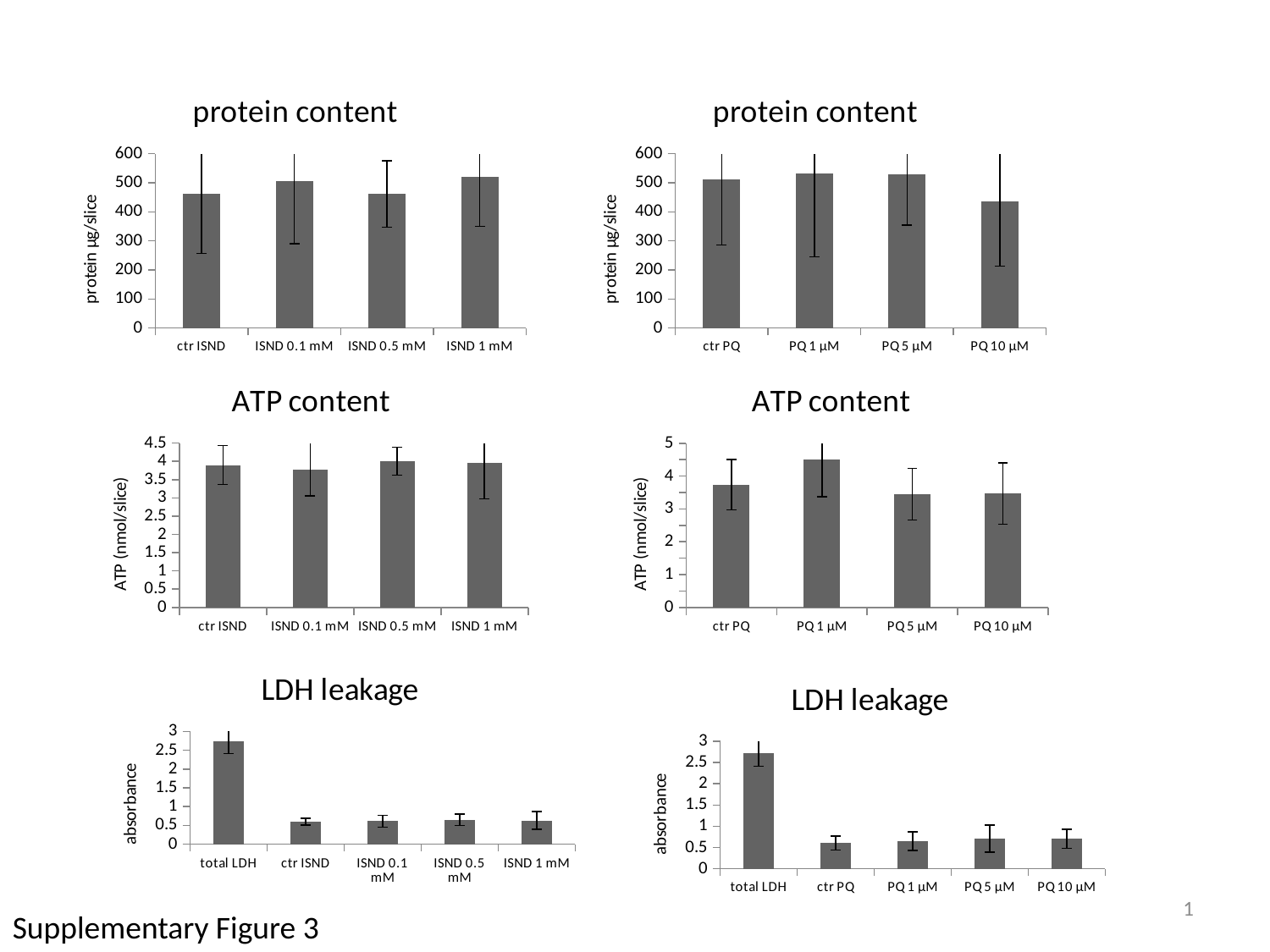

### Chart: protein content
| Category | |
|---|---|
| ctr ISND | 460.9313920000001 |
| ISND 0.1 mM | 505.1388 |
| ISND 0.5 mM | 461.55153600000006 |
| ISND 1 mM | 519.5792960000001 |
### Chart: protein content
| Category | |
|---|---|
| ctr PQ | 511.4900053333334 |
| PQ 1 µM | 532.513728 |
| PQ 5 µM | 530.298928 |
| PQ 10 µM | 434.796752 |
### Chart: ATP content
| Category | |
|---|---|
| ctr ISND | 3.901016 |
| ISND 0.1 mM | 3.780728 |
| ISND 0.5 mM | 4.004168 |
| ISND 1 mM | 3.949064 |
### Chart: ATP content
| Category | |
|---|---|
| ctr PQ | 3.7316720000000005 |
| PQ 1 µM | 4.494875 |
| PQ 5 µM | 3.4511119999999997 |
| PQ 10 µM | 3.466568 |
### Chart: LDH leakage
| Category | |
|---|---|
| total LDH | 2.728 |
| ctr ISND | 0.595 |
| ISND 0.1 mM | 0.6111 |
| ISND 0.5 mM | 0.6491 |
| ISND 1 mM | 0.6313000000000001 |
### Chart: LDH leakage
| Category | |
|---|---|
| total LDH | 2.728 |
| ctr PQ | 0.6057666666666667 |
| PQ 1 µM | 0.6464 |
| PQ 5 µM | 0.7061333333333333 |
| PQ 10 µM | 0.7032999999999999 |1
Supplementary Figure 3
