# Supplementary material for: Treatment of mouse liver slices with cholestatic hepatotoxicants results in down-regulation of Fxr and its target genes
Source: BMC Med Genomics. 2013 Oct 10;6:39. doi: 10.1186/1755-8794-6-39 (PMC3852711; doi:10.1186/1755-8794-6-39)
Supplement: Additional file 4: Table S1 — ANNI gene sets. [file 1755-8794-6-39-S4.doc]

Supplementary Table 1. ANNI gene sets.

| Liver functions | Energy metabolism | Toxicity and drug metabolism | Inflammation | Stress and adaptation | Others |
| --- | --- | --- | --- | --- | --- |
| hepatocyte  Kupffer cells stellate cells  fatty liver  cholestasis  cirrhosis  cholesterol synthesis  liver carcinoma  coagulation  fibrosis  drug metabolism  bile acid metabolism  ABC transporters | lipid metabolism  FA metabolism  mitochondrial diseases  mitochondria  peroxisomes  energy metabolism  glucose metabolism  type 2 diabetes | toxicity  immunocytotoxicity  drug metabolism | Natural Killer cells  lymphocyte  t cells  inflammation  reactome activated TLR4 signalling | biological adaptation to stress  sumoylation  extracellular matrix  protein folding  oxidative stress  hypoxia  apoptosis  necrosis | Morphogenesis  Angiogenesis  tight junctions  Golgi  adipogenesis  osteogenesis |

Gene sets related to diverse hepatic and non-hepatic functions were created in ANNI and were used in GSEA to detect major biological processes affected by treatment with CsA and CPZ. The most upper row informs about the general functional category and the lower cells contain queries used in ANNI to create corresponding gene sets.
