# Supplementary material for: Treatment of mouse liver slices with cholestatic hepatotoxicants results in down-regulation of Fxr and its target genes
Source: BMC Med Genomics. 2013 Oct 10;6:39. doi: 10.1186/1755-8794-6-39 (PMC3852711; doi:10.1186/1755-8794-6-39)
Supplement: Additional file 5: Table S2 — List of primers used for q-PCR. [file 1755-8794-6-39-S5.doc]

Supplementary Table 2. List of primers used for q-PCR.

| Applied Biosystems assay nr | Gene name | Gene symbol |
| --- | --- | --- |
| Mm00517792_m1 | Kruppel-like factor 15 | Klf15 |
| Mm00476075_m1 | bile acid-CoenzymeA:amino acid N-acyltransferase | Baat |
| Mm00445970_m1 | ATP-binding cassette, sub-family G (WHITE), member 8 | Abcg8 |
| Mm00446241_m1 | ATP-binding cassette, sub-family G (WHITE), member 5 | Abcg5 |
