# Supplementary material for: Treatment of mouse liver slices with cholestatic hepatotoxicants results in down-regulation of Fxr and its target genes
Source: BMC Med Genomics. 2013 Oct 10;6:39. doi: 10.1186/1755-8794-6-39 (PMC3852711; doi:10.1186/1755-8794-6-39)
Supplement: Additional file 6: Table S3 — Total pathway analysis. Pathway analysis led to identification of 204 and 125 significantly altered pathways for CsA (A) and CPZ (B) (p < 0.005). Pathways were grouped into functional categories according to MetaCore pathways classification (first column). Each functional category contains pathway name and p value. Additionally number of significantly affected genes and total number of gene in pathways are given. Pathways that were discussed in the paper in details are in bold and underlined. [file 1755-8794-6-39-S6.doc]

**Supplementary Table 3. Total pathway analysis.**

Pathway analysis led to identification of 204 and 125 significantly altered pathways for CsA (A) and CPZ (B) (p<0.005).

Pathways were grouped into functional categories according to MetaCore pathways classification (first column). Each functional category contains pathway name and p value. Additionally number of significantly affected genes and total number of gene in pathways are given. Pathways that were discussed in the paper in details are in bold and underlined.

Supplementary Table 3 A. MetaCore pathway analysis for CsA.

| **Function** | **Pathway** | **p val.** | **Nr of sig altered genes** | **Total nr of genes in a pathway** |
| --- | --- | --- | --- | --- |
| Apoptosis and survival | Anti-apoptotic TNFs/NF-kB/Bcl-2 pathway | 7.018E-08 | [17](javascript:ViewExpDataObj.network_options_func(720);) | 41 |
|  | Anti-apoptotic TNFs/NF-kB/IAP pathway | 2.526E-06 | [12](javascript:ViewExpDataObj.network_options_func(721);) | 27 |
|  | Apoptotic TNF-family pathways | 2.064E-05 | [14](javascript:ViewExpDataObj.network_options_func(728);) | 42 |
|  | APRIL and BAFF signaling | 2.843E-08 | [17](javascript:ViewExpDataObj.network_options_func(2375);) | 39 |
|  | BAD phosphorylation | 1.580E-03 | [11](javascript:ViewExpDataObj.network_options_func(661);) | 42 |
|  | Caspase cascade | 7.287E-07 | [14](javascript:ViewExpDataObj.network_options_func(373);) | 33 |
|  | Ceramides signaling pathway | 1.020E-03 | [11](javascript:ViewExpDataObj.network_options_func(3072);) | 40 |
|  | Cytoplasmic/mitochondrial transport of proapoptotic proteins Bid, Bmf and Bim | 7.333E-06 | [13](javascript:ViewExpDataObj.network_options_func(376);) | 34 |
|  | **Endoplasmic reticulum stress response pathway** | **1.861E-13** | [**26**](javascript:ViewExpDataObj.network_options_func(3092);) | **53** |
|  | FAS signaling cascades | 1.423E-06 | [16](javascript:ViewExpDataObj.network_options_func(418);) | 44 |
|  | HTR1A signaling | 2.221E-03 | [12](javascript:ViewExpDataObj.network_options_func(2947);) | 50 |
|  | Lymphotoxin-beta receptor signaling | 2.539E-11 | [21](javascript:ViewExpDataObj.network_options_func(740);) | 42 |
|  | NGF activation of NF-kB | 2.310E-04 | [10](javascript:ViewExpDataObj.network_options_func(653);) | 29 |
|  | NO synthesis and signaling | 3.692E-05 | [16](javascript:ViewExpDataObj.network_options_func(3270);) | 55 |
|  | p53-dependent apoptosis | 4.084E-05 | [11](javascript:ViewExpDataObj.network_options_func(428);) | 29 |
|  | Regulation of Apoptosis by Mitochondrial Proteins | 1.135E-09 | [17](javascript:ViewExpDataObj.network_options_func(5);) | 33 |
|  | Role of IAP-proteins in apoptosis | 8.351E-05 | [11](javascript:ViewExpDataObj.network_options_func(633);) | 31 |
|  | TNFR1 signaling pathway | 4.014E-10 | [20](javascript:ViewExpDataObj.network_options_func(420);) | 43 |
| Cell adhesion | Chemokines and adhesion | 5.526E-06 | [25](javascript:ViewExpDataObj.network_options_func(716);) | 100 |
|  | **ECM remodeling** | **1.343E-07** | [**19**](javascript:ViewExpDataObj.network_options_func(717);) | **52** |
|  | Plasmin signaling | 1.065E-05 | [13](javascript:ViewExpDataObj.network_options_func(637);) | 35 |
|  | PLAU signaling | 1.919E-04 | [12](javascript:ViewExpDataObj.network_options_func(677);) | 39 |
| Cell cycle | Cell cycle (generic schema) | 4.552E-04 | [8](javascript:ViewExpDataObj.network_options_func(645);) | 21 |
|  | Influence of Ras and Rho proteins on G1/S Transition | 2.214E-05 | [16](javascript:ViewExpDataObj.network_options_func(4583);) | 53 |
|  | Regulation of G1/S transition (part 1) | 2.458E-03 | [10](javascript:ViewExpDataObj.network_options_func(544);) | 38 |
|  | Regulation of G1/S transition (part 2) | 4.605E-04 | [9](javascript:ViewExpDataObj.network_options_func(474);) | 26 |
|  | Normal and pathological TGF-beta-mediated regulation of cell proliferation | 4.961E-06 | [13](javascript:ViewExpDataObj.network_options_func(2930);) | 33 |
| Chemotaxis | CCR4-induced chemotaxis of immune cells | 3.837E-03 | [9](javascript:ViewExpDataObj.network_options_func(2120);) | 34 |
|  | Leukocyte chemotaxis | 1.959E-04 | [18](javascript:ViewExpDataObj.network_options_func(452);) | 75 |
|  | Lipoxin inhibitory action on fMLP-induced neutrophil chemotaxis | 3.453E-03 | [11](javascript:ViewExpDataObj.network_options_func(2728);) | 46 |
| Cytoskeleton remodeling | Cytoskeleton remodeling | 2.434E-06 | [26](javascript:ViewExpDataObj.network_options_func(714);) | 102 |
|  | FAK signaling | 2.415E-03 | [13](javascript:ViewExpDataObj.network_options_func(449);) | 57 |
|  | TGF, WNT and cytoskeletal remodeling | 3.671E-07 | [29](javascript:ViewExpDataObj.network_options_func(715);) | 111 |
| Development | A2A receptor signaling | 5.252E-04 | [12](javascript:ViewExpDataObj.network_options_func(643);) | 43 |
|  | A2B receptor: action via G-protein alpha s | 6.572E-04 | [13](javascript:ViewExpDataObj.network_options_func(482);) | 50 |
|  | A3 receptor signaling | 5.323E-04 | [13](javascript:ViewExpDataObj.network_options_func(644);) | 49 |
|  | Angiotensin activation of ERK | 7.486E-04 | [10](javascript:ViewExpDataObj.network_options_func(437);) | 33 |
|  | Angiotensin signaling via PYK2 | 5.252E-04 | [12](javascript:ViewExpDataObj.network_options_func(438);) | 43 |
|  | Beta-adrenergic receptors transactivation of EGFR | 4.927E-04 | [11](javascript:ViewExpDataObj.network_options_func(2433);) | 37 |
|  | Cross-talk between VEGF and Angiopoietin 1 signaling pathways | 4.605E-04 | [9](javascript:ViewExpDataObj.network_options_func(534);) | 26 |
|  | Delta-type opioid receptor mediated cardioprotection | 4.927E-04 | [11](javascript:ViewExpDataObj.network_options_func(2666);) | 37 |
|  | Dopamine D2 receptor transactivation of EGFR | 1.268E-03 | [8](javascript:ViewExpDataObj.network_options_func(2457);) | 24 |
|  | EGFR signaling pathway | 2.169E-03 | [14](javascript:ViewExpDataObj.network_options_func(443);) | 63 |
|  | EGFR signaling via PIP3 | 9.218E-04 | [8](javascript:ViewExpDataObj.network_options_func(692);) | 23 |
|  | EGFR signaling via small GTPases | 3.078E-03 | [9](javascript:ViewExpDataObj.network_options_func(704);) | 33 |
|  | Endothelin-1/EDNRA signaling | 1.010E-06 | [18](javascript:ViewExpDataObj.network_options_func(2255);) | 53 |
|  | Endothelin-1/EDNRA transactivation of EGFR | 2.705E-04 | [13](javascript:ViewExpDataObj.network_options_func(2254);) | 46 |
|  | ERBB-family signaling | 3.027E-03 | [10](javascript:ViewExpDataObj.network_options_func(535);) | 39 |
|  | FGF2-dependent induction of EMT | 1.856E-03 | [7](javascript:ViewExpDataObj.network_options_func(3020);) | 20 |
|  | FGF-family signaling | 9.821E-04 | [13](javascript:ViewExpDataObj.network_options_func(549);) | 52 |
|  | FGFR signaling pathway | 2.870E-05 | [16](javascript:ViewExpDataObj.network_options_func(444);) | 54 |
|  | Flt3 signaling | 3.740E-05 | [14](javascript:ViewExpDataObj.network_options_func(2236);) | 44 |
|  | Gastrin in cell growth and proliferation | 1.256E-05 | [18](javascript:ViewExpDataObj.network_options_func(3134);) | 62 |
|  | G-CSF signaling | 1.845E-03 | [12](javascript:ViewExpDataObj.network_options_func(6413);) | 49 |
|  | Glucocorticoid receptor signaling | 1.268E-03 | [8](javascript:ViewExpDataObj.network_options_func(410);) | 24 |
|  | Growth hormone signaling via PI3K/AKT and MAPK cascades | 4.142E-04 | [12](javascript:ViewExpDataObj.network_options_func(2253);) | 42 |
|  | Hedgehog and PTH signaling pathways in bone and cartilage development | 1.577E-03 | [10](javascript:ViewExpDataObj.network_options_func(553);) | 36 |
|  | HGF signaling pathway | 1.246E-07 | [18](javascript:ViewExpDataObj.network_options_func(530);) | 47 |
|  | HGF-dependent inhibition of TGF-beta-induced EMT | 3.837E-03 | [9](javascript:ViewExpDataObj.network_options_func(3019);) | 34 |
|  | IGF-1 receptor signaling | 2.778E-04 | [14](javascript:ViewExpDataObj.network_options_func(540);) | 52 |
|  | Inhibition of angiogenesis by PEDF | 4.283E-04 | [10](javascript:ViewExpDataObj.network_options_func(5591);) | 31 |
|  | Keratinocyte differentiation | 6.342E-04 | [14](javascript:ViewExpDataObj.network_options_func(4613);) | 56 |
|  | Leptin signaling via JAK/STAT and MAPK cascades | 9.348E-08 | [13](javascript:ViewExpDataObj.network_options_func(718);) | 25 |
|  | Leptin signaling via PI3K-dependent pathway | 3.416E-04 | [13](javascript:ViewExpDataObj.network_options_func(719);) | 47 |
|  | Mu-type opioid receptor regulation of proliferation | 3.792E-03 | [8](javascript:ViewExpDataObj.network_options_func(2424);) | 28 |
|  | Mu-type opioid receptor signaling | 2.458E-03 | [10](javascript:ViewExpDataObj.network_options_func(2453);) | 38 |
|  | Neurotrophin family signaling | 3.697E-03 | [10](javascript:ViewExpDataObj.network_options_func(636);) | 40 |
|  | PDGF signaling via STATs and NF-kB | 1.163E-04 | [11](javascript:ViewExpDataObj.network_options_func(635);) | 32 |
|  | PEDF signaling | 1.507E-11 | [23](javascript:ViewExpDataObj.network_options_func(4864);) | 49 |
|  | PIP3 signaling in cardiac myocytes | 3.894E-06 | [16](javascript:ViewExpDataObj.network_options_func(701);) | 47 |
|  | Regulation of epithelial-to-mesenchymal transition (EMT) | 9.294E-09 | [23](javascript:ViewExpDataObj.network_options_func(3018);) | 64 |
|  | Role of HDAC and calcium/calmodulin-dependent kinase (CaMK) in control of skeletal myogenesis | 1.431E-03 | [13](javascript:ViewExpDataObj.network_options_func(440);) | 54 |
|  | Role of IL-8 in angiogenesis | 2.955E-03 | [14](javascript:ViewExpDataObj.network_options_func(3051);) | 65 |
|  | S1P1 signaling pathway | 2.370E-03 | [11](javascript:ViewExpDataObj.network_options_func(2809);) | 44 |
|  | S1P3 receptor signaling pathway | 1.942E-03 | [11](javascript:ViewExpDataObj.network_options_func(2951);) | 43 |
|  | TGF-beta receptor signaling | 9.676E-06 | [16](javascript:ViewExpDataObj.network_options_func(475);) | 50 |
|  | TGF-beta-dependent induction of EMT via MAPK | 3.709E-10 | [21](javascript:ViewExpDataObj.network_options_func(2997);) | 47 |
|  | TGF-beta-dependent induction of EMT via RhoA, PI3K and ILK. | 1.018E-03 | [12](javascript:ViewExpDataObj.network_options_func(2995);) | 46 |
|  | TGF-beta-dependent induction of EMT via SMADs | 1.065E-05 | [13](javascript:ViewExpDataObj.network_options_func(2996);) | 35 |
|  | Thrombopoetin signaling via JAK-STAT pathway | 3.443E-03 | [7](javascript:ViewExpDataObj.network_options_func(469);) | 22 |
|  | Thrombopoietin-regulated cell processes | 1.048E-05 | [15](javascript:ViewExpDataObj.network_options_func(631);) | 45 |
|  | Thrombospondin-1 signaling | 3.792E-03 | [8](javascript:ViewExpDataObj.network_options_func(3106);) | 28 |
|  | VEGF signaling and activation | 5.252E-04 | [12](javascript:ViewExpDataObj.network_options_func(539);) | 43 |
|  | VEGF signaling via VEGFR2 - generic cascades | 8.971E-06 | [22](javascript:ViewExpDataObj.network_options_func(533);) | 84 |
|  | VEGF-family signaling | 7.367E-05 | [13](javascript:ViewExpDataObj.network_options_func(445);) | 41 |
|  | WNT signaling pathway. Part 2 | 3.448E-04 | [14](javascript:ViewExpDataObj.network_options_func(516);) | 53 |
| DNA damage | ATM/ATR regulation of G1/S checkpoint | 2.443E-03 | [9](javascript:ViewExpDataObj.network_options_func(426);) | 32 |
|  | Role of Brca1 and Brca2 in DNA repair | 1.484E-03 | [9](javascript:ViewExpDataObj.network_options_func(427);) | 30 |
| G-protein signaling | G-Protein alpha-q signaling cascades | 9.707E-04 | [10](javascript:ViewExpDataObj.network_options_func(639);) | 34 |
|  | Proinsulin C-peptide signaling | 2.778E-04 | [14](javascript:ViewExpDataObj.network_options_func(2815);) | 52 |
|  | Ras family GTPases in kinase cascades (scheme) | 2.265E-03 | [8](javascript:ViewExpDataObj.network_options_func(379);) | 26 |
|  | Regulation of p38 and JNK signaling mediated by G-proteins | 1.919E-04 | [12](javascript:ViewExpDataObj.network_options_func(455);) | 39 |
| Immune response | HIV-1 signaling via CCR5 in macrophages and T lymphocytes | 3.027E-03 | [10](javascript:ViewExpDataObj.network_options_func(3073);) | 39 |
|  | IL-1 beta-dependent CFTR expression | 4.456E-05 | [8](javascript:ViewExpDataObj.network_options_func(2943);) | 16 |
|  | Cytokine production by Th17 cells in CF (Mouse model) | 2.617E-07 | [18](javascript:ViewExpDataObj.network_options_func(2695);) | 49 |
|  | CCR3 signaling in eosinophils | 8.244E-04 | [17](javascript:ViewExpDataObj.network_options_func(736);) | 77 |
|  | IFN gamma signaling pathway | 4.251E-04 | [14](javascript:ViewExpDataObj.network_options_func(432);) | 54 |
|  | Bacterial infections in normal airways | 6.572E-04 | [13](javascript:ViewExpDataObj.network_options_func(2694);) | 50 |
|  | BCR pathway | 1.431E-03 | [13](javascript:ViewExpDataObj.network_options_func(655);) | 54 |
|  | C5a signaling | 6.440E-08 | [19](javascript:ViewExpDataObj.network_options_func(6453);) | 50 |
|  | CCR5 signaling in macrophages and T lymphocytes | 9.246E-04 | [14](javascript:ViewExpDataObj.network_options_func(3071);) | 58 |
|  | CD137 signaling in immune cell | 1.028E-08 | [15](javascript:ViewExpDataObj.network_options_func(3053);) | 29 |
|  | CD16 signaling in NK cells | 1.930E-03 | [15](javascript:ViewExpDataObj.network_options_func(2249);) | 69 |
|  | CD28 signaling | 4.251E-04 | [14](javascript:ViewExpDataObj.network_options_func(620);) | 54 |
|  | CD40 signaling | 6.842E-08 | [22](javascript:ViewExpDataObj.network_options_func(3074);) | 65 |
|  | CXCR4 signaling via second messenger | 2.156E-04 | [11](javascript:ViewExpDataObj.network_options_func(613);) | 34 |
|  | Delta-type opioid receptor signaling in T-cells | 2.310E-04 | [10](javascript:ViewExpDataObj.network_options_func(2665);) | 29 |
|  | Fc epsilon RI pathway | 3.692E-05 | [16](javascript:ViewExpDataObj.network_options_func(566);) | 55 |
|  | Function of MEF2 in T lymphocytes | 2.221E-03 | [12](javascript:ViewExpDataObj.network_options_func(541);) | 50 |
|  | Gastrin in inflammatory response | 1.724E-09 | [25](javascript:ViewExpDataObj.network_options_func(3136);) | 69 |
|  | Histamine H1 receptor signaling in immune response | 2.945E-08 | [19](javascript:ViewExpDataObj.network_options_func(2434);) | 48 |
|  | Histamine signaling in dendritic cells | 1.766E-04 | [14](javascript:ViewExpDataObj.network_options_func(2438);) | 50 |
|  | HMGB1 release from the cell | 2.783E-06 | [15](javascript:ViewExpDataObj.network_options_func(6113);) | 41 |
|  | HMGB1/RAGE signaling pathway | 1.419E-11 | [24](javascript:ViewExpDataObj.network_options_func(6111);) | 53 |
|  | HMGB1/TLR signaling pathway | 3.869E-07 | [15](javascript:ViewExpDataObj.network_options_func(6112);) | 36 |
|  | **HSP60 and HSP70/ TLR signaling pathway** | **1.755E-10** | [**23**](javascript:ViewExpDataObj.network_options_func(6429);) | **54** |
|  | ICOS pathway in T-helper cell | 3.453E-03 | [11](javascript:ViewExpDataObj.network_options_func(619);) | 46 |
|  | **IL-1 signaling pathway** | **7.874E-11** | [**21**](javascript:ViewExpDataObj.network_options_func(658);) | **44** |
|  | IL-12-induced IFN-gamma production | 5.225E-08 | [16](javascript:ViewExpDataObj.network_options_func(2747);) | 36 |
|  | IL-13 signaling via JAK-STAT | 6.602E-04 | [12](javascript:ViewExpDataObj.network_options_func(4361);) | 44 |
|  | IL-15 signaling | 5.031E-06 | [19](javascript:ViewExpDataObj.network_options_func(3054);) | 64 |
|  | IL-17 signaling pathways | 2.154E-09 | [23](javascript:ViewExpDataObj.network_options_func(3088);) | 60 |
|  | IL-2 activation and signaling pathway | 5.323E-04 | [13](javascript:ViewExpDataObj.network_options_func(430);) | 49 |
|  | IL-22 signaling pathway | 3.837E-03 | [9](javascript:ViewExpDataObj.network_options_func(522);) | 34 |
|  | IL-23 signaling pathway | 3.288E-04 | [9](javascript:ViewExpDataObj.network_options_func(2748);) | 25 |
|  | IL-4 - antiapoptotic action | 1.704E-07 | [14](javascript:ViewExpDataObj.network_options_func(659);) | 30 |
|  | IL-4 signaling pathway | 6.602E-04 | [12](javascript:ViewExpDataObj.network_options_func(431);) | 44 |
|  | IL-5 signalling | 2.370E-03 | [11](javascript:ViewExpDataObj.network_options_func(3262);) | 44 |
|  | IL-7 signaling in T lymphocytes | 2.458E-03 | [10](javascript:ViewExpDataObj.network_options_func(3173);) | 38 |
|  | IL-9 signaling pathway | 3.869E-07 | [15](javascript:ViewExpDataObj.network_options_func(523);) | 36 |
|  | Inflammasome in inflammatory response | 2.475E-07 | [15](javascript:ViewExpDataObj.network_options_func(6121);) | 35 |
|  | Inhibitory action of Lipoxins on pro-inflammatory TNF-alpha signaling | 6.503E-05 | [14](javascript:ViewExpDataObj.network_options_func(2727);) | 46 |
|  | Innate immune response to RNA viral infection | 8.538E-04 | [9](javascript:ViewExpDataObj.network_options_func(3076);) | 28 |
|  | MIF in innate immunity response | 3.105E-07 | [16](javascript:ViewExpDataObj.network_options_func(519);) | 40 |
|  | MIF-mediated glucocorticoid regulation | 1.177E-08 | [13](javascript:ViewExpDataObj.network_options_func(2022);) | 22 |
|  | NF-AT signaling and leukocyte interactions | 3.453E-03 | [11](javascript:ViewExpDataObj.network_options_func(2227);) | 46 |
|  | Oncostatin M signaling via MAPK in human cells | 3.767E-06 | [14](javascript:ViewExpDataObj.network_options_func(2204);) | 37 |
|  | Oncostatin M signaling via MAPK in mouse cells | 1.065E-05 | [13](javascript:ViewExpDataObj.network_options_func(2205);) | 35 |
|  | PGE2 in immune and neuroendocrine system interactions | 1.654E-04 | [13](javascript:ViewExpDataObj.network_options_func(2387);) | 44 |
|  | PGE2 signaling in immune response | 2.124E-04 | [13](javascript:ViewExpDataObj.network_options_func(2388);) | 45 |
|  | Regulation of T cell function by CTLA-4 | 3.786E-04 | [11](javascript:ViewExpDataObj.network_options_func(3056);) | 36 |
|  | Role of HMGB1 in dendritic cell maturation and migration | 3.003E-07 | [13](javascript:ViewExpDataObj.network_options_func(6114);) | 27 |
|  | Role of integrins in NK cells cytotoxicity | 2.973E-05 | [13](javascript:ViewExpDataObj.network_options_func(2228);) | 38 |
|  | Signaling pathway mediated by IL-6 and IL-1 | 9.573E-06 | [12](javascript:ViewExpDataObj.network_options_func(478);) | 30 |
|  | Th1 and Th2 cell differentiation | 1.020E-03 | [11](javascript:ViewExpDataObj.network_options_func(3082);) | 40 |
|  | Th17 cell differentiation | 2.875E-04 | [11](javascript:ViewExpDataObj.network_options_func(3268);) | 35 |
|  | TLR signaling pathways | 2.681E-07 | [19](javascript:ViewExpDataObj.network_options_func(558);) | 54 |
|  | TLR3 and TLR4 induce TICAM1-specific signaling pathway | 1.856E-03 | [7](javascript:ViewExpDataObj.network_options_func(559);) | 20 |
|  | TREM1 signaling pathway | 4.998E-08 | [21](javascript:ViewExpDataObj.network_options_func(2955);) | 59 |
|  | Blood coagulation_Blood coagulation | 3.027E-03 | [10](javascript:ViewExpDataObj.network_options_func(3104);) | 39 |
| Protein folding &processing | Angiotensin system maturation \ Human version | 5.252E-04 | [12](javascript:ViewExpDataObj.network_options_func(2647);) | 43 |
|  | Angiotensin system maturation \ Rodent version | 1.524E-03 | [12](javascript:ViewExpDataObj.network_options_func(2648);) | 48 |
|  | POMC processing | 1.539E-10 | [17](javascript:ViewExpDataObj.network_options_func(5358);) | 30 |
|  | Putative SUMO-1 pathway | 1.134E-03 | [9](javascript:ViewExpDataObj.network_options_func(699);) | 29 |
| Regulation of metabolism | Putative pathways for stimulation of fat cell differentiation by Bisphenol A | 5.701E-04 | [10](javascript:ViewExpDataObj.network_options_func(5030);) | 32 |
|  | FXR-dependent negative-feedback regulation of bile acids concentration | 8.351E-05 | [11](javascript:ViewExpDataObj.network_options_func(3090);) | 31 |
|  | Insulin regulation of fatty acid methabolism | 1.719E-03 | [18](javascript:ViewExpDataObj.network_options_func(726);) | 89 |
|  | Insulin regulation of glycogen metabolism | 6.342E-04 | [14](javascript:ViewExpDataObj.network_options_func(725);) | 56 |
|  | Insulin signaling:generic cascades | 4.126E-03 | [11](javascript:ViewExpDataObj.network_options_func(724);) | 47 |
|  | PPAR regulation of lipid metabolism | 1.580E-03 | [11](javascript:ViewExpDataObj.network_options_func(632);) | 42 |
|  | **RXR-dependent regulation of lipid metabolism via PPAR, RAR and VDR** | **5.894E-05** | [**11**](javascript:ViewExpDataObj.network_options_func(413);) | **30** |
|  | **Bile acids regulation of glucose and lipid metabolism via FXR** | **7.256E-14** | [**22**](javascript:ViewExpDataObj.network_options_func(3089);) | **37** |
|  | Role of Adiponectin in regulation of metabolism | 1.620E-07 | [17](javascript:ViewExpDataObj.network_options_func(4733);) | 43 |
|  | **FXR-regulated cholesterol and bile acids cellular transport** | **6.839E-07** | [**16**](javascript:ViewExpDataObj.network_options_func(3091);) | **42** |
| Reproduction | Reproduction_GnRH signaling | 8.757E-06 | [20](javascript:ViewExpDataObj.network_options_func(3137);) | 72 |
| Pathogenesis of obesity | Role of adipose tissue hypoxia in obesity and type 2 diabetes | 1.812E-09 | [16](javascript:ViewExpDataObj.network_options_func(4724);) | 30 |
|  | Role of ER stress in obesity and type 2 diabetes | 3.555E-09 | [21](javascript:ViewExpDataObj.network_options_func(4723);) | 52 |
|  | Role of free fatty acids in obesity and type 2 diabetes | 2.253E-10 | [21](javascript:ViewExpDataObj.network_options_func(4633);) | 46 |
|  | Role of Ghrelin in activation eating behavior in obesity | 3.697E-03 | [10](javascript:ViewExpDataObj.network_options_func(5062);) | 40 |
|  | Role of GIP in pathogenesis of type 2 diabetes | 2.705E-04 | [13](javascript:ViewExpDataObj.network_options_func(5584);) | 46 |
|  | Role of IL-6 in obesity and type 2 diabetes in adipocytes | 3.294E-06 | [13](javascript:ViewExpDataObj.network_options_func(4911);) | 32 |
|  | Role of inflammasome in macrophages, adipocytes and pancreatic beta cells in type 2 diabetes | 1.453E-05 | [9](javascript:ViewExpDataObj.network_options_func(6101);) | 18 |
|  | Role of Insulin in regulation of eating behavior in obesity | 9.932E-07 | [16](javascript:ViewExpDataObj.network_options_func(5228);) | 43 |
|  | Role of Leptin in regulation of eating behavior in obesity | 1.569E-04 | [9](javascript:ViewExpDataObj.network_options_func(5218);) | 23 |
|  | Role of TNF-alpha in type 2 diabetes in skeletal muscle cells | 6.839E-07 | [16](javascript:ViewExpDataObj.network_options_func(4912);) | 42 |
|  | Role of TNF-alpha, IL-1beta and IL-6 in development of obesity and type 2 diabetes in liver | 5.156E-13 | [24](javascript:ViewExpDataObj.network_options_func(4910);) | 47 |
|  | Selective Insulin resistance in type 2 diabetes in liver | 3.940E-12 | [19](javascript:ViewExpDataObj.network_options_func(5349);) | 32 |
|  | VLDL, LDL dyslipidemia in type 2 diabetes and metabolic syndrome X | 8.080E-04 | [11](javascript:ViewExpDataObj.network_options_func(6369);) | 39 |
|  | TNF-alpha and IL-1 beta induce hyperglycemia in obesity and type 2 diabetes in adipocytes | 3.480E-11 | [19](javascript:ViewExpDataObj.network_options_func(4789);) | 35 |
|  | TNF-alpha, IL-1 beta induce dyslipidemia and inflammation in obesity and type 2 diabetes in adipocytes | 1.423E-06 | [16](javascript:ViewExpDataObj.network_options_func(4790);) | 44 |
|  | Chemokines in inflammation in adipose tissue and liver in obesity, type 2 diabetes and metabolic syndrome X | 8.783E-12 | [23](javascript:ViewExpDataObj.network_options_func(13015);) | 48 |
|  | Chylomicron dyslipidemia in type 2 diabetes and metabolic syndrome X | 5.509E-05 | [13](javascript:ViewExpDataObj.network_options_func(13016);) | 40 |
|  | Fenofibrate in treatment of type 2 diabetes and metabolic syndrome X | 3.288E-04 | [9](javascript:ViewExpDataObj.network_options_func(6252);) | 25 |
|  | Adiponectin in pathogenesis of type 2 diabetes | 6.289E-06 | [12](javascript:ViewExpDataObj.network_options_func(5951);) | 29 |
|  | GLP-1 in beta cell apoptosis in type 2 diabetes | 1.816E-07 | [18](javascript:ViewExpDataObj.network_options_func(5583);) | 48 |
|  | Glucagon-induced glucose upregulation in type 2 diabetes in liver | 1.694E-05 | [16](javascript:ViewExpDataObj.network_options_func(5195);) | 52 |
|  | Pioglitazone and Rosiglitazone in treatment of type 2 diabetes and metabolic syndrome X | 1.706E-07 | [13](javascript:ViewExpDataObj.network_options_func(6251);) | 26 |
|  | Processes in pathogenesis of Diabetes mellitus II, Insulinoresistance and Obesity | 1.272E-04 | [8](javascript:ViewExpDataObj.network_options_func(11157);) | 18 |
| Signal transduction | AKT signaling | 9.932E-07 | [16](javascript:ViewExpDataObj.network_options_func(554);) | 43 |
|  | IP3 signaling | 1.393E-04 | [14](javascript:ViewExpDataObj.network_options_func(557);) | 49 |
|  | JNK pathway | 3.949E-06 | [15](javascript:ViewExpDataObj.network_options_func(526);) | 42 |
|  | PTEN pathway | 6.503E-05 | [14](javascript:ViewExpDataObj.network_options_func(676);) | 46 |
|  | Some pathways of EMT in cancer cells | 2.711E-06 | [17](javascript:ViewExpDataObj.network_options_func(3017);) | 51 |
|  | Androgen Receptor nuclear signaling | 2.870E-03 | [11](javascript:ViewExpDataObj.network_options_func(2202);) | 45 |
|  | **NF-kB signaling pathway** | **4.185E-11** | [**20**](javascript:ViewExpDataObj.network_options_func(411);) | **39** |
|  | P53 signaling pathway | 1.326E-06 | [15](javascript:ViewExpDataObj.network_options_func(412);) | 39 |
|  | PPAR Pathway | 1.603E-05 | [18](javascript:ViewExpDataObj.network_options_func(546);) | 63 |
|  | Receptor-mediated HIF regulation | 1.919E-04 | [12](javascript:ViewExpDataObj.network_options_func(416);) | 39 |
|  | Role of Akt in hypoxia induced HIF1 activation | 6.325E-04 | [9](javascript:ViewExpDataObj.network_options_func(448);) | 27 |
|  | Role of AP-1 in regulation of cellular metabolism | 1.454E-04 | [12](javascript:ViewExpDataObj.network_options_func(434);) | 38 |
|  | Role of VDR in regulation of genes involved in osteoporosis | 4.527E-03 | [13](javascript:ViewExpDataObj.network_options_func(669);) | 61 |
|  | Regulation of EIF2 activity | 8.080E-04 | [11](javascript:ViewExpDataObj.network_options_func(497);) | 39 |
|  | Regulation of EIF4F activity | 2.214E-05 | [16](javascript:ViewExpDataObj.network_options_func(496);) | 53 |
|  | Non-genomic (rapid) action of Androgen Receptor | 1.020E-03 | [11](javascript:ViewExpDataObj.network_options_func(2203);) | 40 |

Supplementary Table 3 B. MetaCore pathway analysis for CPZ.

| **Function** | **Pathway** | **p val.** | **Nr of sig altered genes** | **Total nr of genes in a pathway** |
| --- | --- | --- | --- | --- |
| Apoptosis and survival | Apoptotic Activin A signaling | 3.050E-03 | [6](javascript:ViewExpDataObj.network_options_func(2476);) | 25 |
|  | BAD phosphorylation | 7.184E-04 | [9](javascript:ViewExpDataObj.network_options_func(661);) | 42 |
|  | Caspase cascade | 5.906E-04 | [8](javascript:ViewExpDataObj.network_options_func(373);) | 33 |
|  | HTR1A signaling | 2.630E-03 | [9](javascript:ViewExpDataObj.network_options_func(2947);) | 50 |
|  | NO synthesis and signaling | 1.428E-03 | [10](javascript:ViewExpDataObj.network_options_func(3270);) | 55 |
|  | Role of CDK5 in neuronal death and survival | 3.549E-03 | [7](javascript:ViewExpDataObj.network_options_func(2374);) | 34 |
| Cell adhesion | Cell-matrix glycoconjugates | 1.595E-03 | [8](javascript:ViewExpDataObj.network_options_func(741);) | 38 |
|  | Chemokines and adhesion | 1.560E-06 | [20](javascript:ViewExpDataObj.network_options_func(716);) | 100 |
|  | **ECM remodeling** | **4.177E-09** | [**17**](javascript:ViewExpDataObj.network_options_func(717);) | **52** |
|  | Ephrin signaling | 1.215E-03 | [9](javascript:ViewExpDataObj.network_options_func(649);) | 45 |
|  | Integrin-mediated cell adhesion and migration | 1.959E-03 | [9](javascript:ViewExpDataObj.network_options_func(450);) | 48 |
|  | Plasmin signaling | 9.001E-04 | [8](javascript:ViewExpDataObj.network_options_func(637);) | 35 |
|  | PLAU signaling | 2.220E-07 | [13](javascript:ViewExpDataObj.network_options_func(677);) | 39 |
|  | Tight junctions | 1.097E-03 | [8](javascript:ViewExpDataObj.network_options_func(1496);) | 36 |
| Cytoskeleton remodeling | Cytoskeleton remodeling | 5.152E-07 | [21](javascript:ViewExpDataObj.network_options_func(714);) | 102 |
|  | TGF, WNT and cytoskeletal remodeling | 1.303E-07 | [23](javascript:ViewExpDataObj.network_options_func(715);) | 111 |
| Development | ACM2 and ACM4 activation of ERK | 8.610E-04 | [9](javascript:ViewExpDataObj.network_options_func(2516);) | 43 |
|  | Activation of Erk by ACM1, ACM3 and ACM5 | 4.210E-03 | [8](javascript:ViewExpDataObj.network_options_func(2518);) | 44 |
|  | Activation of ERK by Kappa-type opioid receptor | 4.964E-03 | [7](javascript:ViewExpDataObj.network_options_func(2552);) | 36 |
|  | Angiotensin activation of ERK | 2.969E-03 | [7](javascript:ViewExpDataObj.network_options_func(437);) | 33 |
|  | Beta-adrenergic receptors transactivation of EGFR | 1.328E-03 | [8](javascript:ViewExpDataObj.network_options_func(2433);) | 37 |
|  | CNTF receptor signaling | 7.323E-04 | [8](javascript:ViewExpDataObj.network_options_func(2231);) | 34 |
|  | Dopamine D2 receptor transactivation of EGFR | 3.854E-04 | [7](javascript:ViewExpDataObj.network_options_func(2457);) | 24 |
|  | EGFR signaling pathway | 4.056E-03 | [10](javascript:ViewExpDataObj.network_options_func(443);) | 63 |
|  | EGFR signaling via PIP3 | 2.878E-04 | [7](javascript:ViewExpDataObj.network_options_func(692);) | 23 |
|  | EGFR signaling via small GTPases | 2.969E-03 | [7](javascript:ViewExpDataObj.network_options_func(704);) | 33 |
|  | Endothelin-1/EDNRA signaling | 3.963E-03 | [9](javascript:ViewExpDataObj.network_options_func(2255);) | 53 |
|  | EPO-induced Jak-STAT pathway | 4.212E-03 | [7](javascript:ViewExpDataObj.network_options_func(737);) | 35 |
|  | ERBB-family signaling | 1.731E-06 | [12](javascript:ViewExpDataObj.network_options_func(535);) | 39 |
|  | ERK5 in cell proliferation and neuronal survival | 1.926E-03 | [6](javascript:ViewExpDataObj.network_options_func(3231);) | 23 |
|  | FGF-family signaling | 8.419E-06 | [13](javascript:ViewExpDataObj.network_options_func(549);) | 52 |
|  | FGFR signaling pathway | 4.509E-03 | [9](javascript:ViewExpDataObj.network_options_func(444);) | 54 |
|  | G-CSF-induced myeloid differentiation | 1.655E-03 | [7](javascript:ViewExpDataObj.network_options_func(6415);) | 30 |
|  | Growth hormone signaling via PI3K/AKT and MAPK cascades | 7.184E-04 | [9](javascript:ViewExpDataObj.network_options_func(2253);) | 42 |
|  | HGF signaling pathway | 2.463E-06 | [13](javascript:ViewExpDataObj.network_options_func(530);) | 47 |
|  | HGF-dependent inhibition of TGF-beta-induced EMT | 1.300E-04 | [9](javascript:ViewExpDataObj.network_options_func(3019);) | 34 |
|  | IGF-1 receptor signaling | 3.471E-03 | [9](javascript:ViewExpDataObj.network_options_func(540);) | 52 |
|  | Keratinocyte differentiation | 1.648E-03 | [10](javascript:ViewExpDataObj.network_options_func(4613);) | 56 |
|  | Leptin signaling via JAK/STAT and MAPK cascades | 8.179E-07 | [10](javascript:ViewExpDataObj.network_options_func(718);) | 25 |
|  | Leptin signaling via PI3K-dependent pathway | 8.049E-05 | [11](javascript:ViewExpDataObj.network_options_func(719);) | 47 |
|  | Ligand-independent activation of ESR1 and ESR2 | 2.014E-07 | [14](javascript:ViewExpDataObj.network_options_func(2210);) | 45 |
|  | Melanocyte development and pigmentation | 5.553E-04 | [10](javascript:ViewExpDataObj.network_options_func(4863);) | 49 |
|  | Membrane-bound ESR1: interaction with growth factors signaling | 9.131E-06 | [12](javascript:ViewExpDataObj.network_options_func(2211);) | 45 |
|  | Neurotrophin family signaling | 2.256E-03 | [8](javascript:ViewExpDataObj.network_options_func(636);) | 40 |
|  | NOTCH-induced EMT | 6.361E-04 | [6](javascript:ViewExpDataObj.network_options_func(3023);) | 19 |
|  | PIP3 signaling in cardiac myocytes | 3.900E-04 | [10](javascript:ViewExpDataObj.network_options_func(701);) | 47 |
|  | Prolactin receptor signaling | 2.168E-03 | [10](javascript:ViewExpDataObj.network_options_func(545);) | 58 |
|  | Regulation of epithelial-to-mesenchymal transition (EMT) | 2.139E-08 | [18](javascript:ViewExpDataObj.network_options_func(3018);) | 64 |
|  | Role of HDAC and calcium/calmodulin-dependent kinase (CaMK) in control of skeletal myogenesis | 4.509E-03 | [9](javascript:ViewExpDataObj.network_options_func(440);) | 54 |
|  | Slit-Robo signaling | 1.655E-03 | [7](javascript:ViewExpDataObj.network_options_func(2121);) | 30 |
|  | TGF-beta receptor signaling | 1.464E-04 | [11](javascript:ViewExpDataObj.network_options_func(475);) | 50 |
|  | TGF-beta-dependent induction of EMT via MAPK | 1.485E-05 | [12](javascript:ViewExpDataObj.network_options_func(2997);) | 47 |
|  | TGF-beta-dependent induction of EMT via SMADs | 3.729E-06 | [11](javascript:ViewExpDataObj.network_options_func(2996);) | 35 |
|  | Thrombopoietin-regulated cell processes | 2.678E-04 | [10](javascript:ViewExpDataObj.network_options_func(631);) | 45 |
|  | Transcription regulation of granulocyte development | 2.465E-03 | [7](javascript:ViewExpDataObj.network_options_func(458);) | 32 |
|  | VEGF-family signaling | 5.956E-04 | [9](javascript:ViewExpDataObj.network_options_func(445);) | 41 |
|  | WNT signaling pathway. Part 2 | 1.061E-03 | [10](javascript:ViewExpDataObj.network_options_func(516);) | 53 |
| G-protein signaling | K-RAS regulation pathway | 5.079E-04 | [7](javascript:ViewExpDataObj.network_options_func(399);) | 25 |
|  | N-RAS regulation pathway | 5.906E-04 | [8](javascript:ViewExpDataObj.network_options_func(400);) | 33 |
| Immune response | Blood coagulation | 4.012E-04 | [9](javascript:ViewExpDataObj.network_options_func(3104);) | 39 |
|  | Sialic-acid receptors (Siglecs) signaling | 4.345E-03 | [4](javascript:ViewExpDataObj.network_options_func(732);) | 12 |
|  | C5a signaling | 2.923E-05 | [12](javascript:ViewExpDataObj.network_options_func(6453);) | 50 |
|  | CD16 signaling in NK cells | 2.538E-03 | [11](javascript:ViewExpDataObj.network_options_func(2249);) | 69 |
|  | CXCR4 signaling via second messenger | 3.549E-03 | [7](javascript:ViewExpDataObj.network_options_func(613);) | 34 |
|  | Gastrin in inflammatory response | 2.538E-03 | [11](javascript:ViewExpDataObj.network_options_func(3136);) | 69 |
|  | HMGB1/RAGE signaling pathway | 3.963E-03 | [9](javascript:ViewExpDataObj.network_options_func(6111);) | 53 |
|  | IFN alpha/beta signaling pathway | 3.854E-04 | [7](javascript:ViewExpDataObj.network_options_func(429);) | 24 |
|  | IL-1 signaling pathway | 4.210E-03 | [8](javascript:ViewExpDataObj.network_options_func(658);) | 44 |
|  | IL-13 signaling via PI3K-ERK | 2.630E-03 | [9](javascript:ViewExpDataObj.network_options_func(4518);) | 50 |
|  | IL-17 signaling pathways | 2.812E-03 | [10](javascript:ViewExpDataObj.network_options_func(3088);) | 60 |
|  | IL-2 activation and signaling pathway | 1.207E-04 | [11](javascript:ViewExpDataObj.network_options_func(430);) | 49 |
|  | IL-3 activation and signaling pathway | 7.988E-06 | [10](javascript:ViewExpDataObj.network_options_func(657);) | 31 |
|  | IL-4 - antiapoptotic action | 2.921E-04 | [8](javascript:ViewExpDataObj.network_options_func(659);) | 30 |
|  | IL-4 signaling pathway | 4.210E-03 | [8](javascript:ViewExpDataObj.network_options_func(431);) | 44 |
|  | IL-5 signalling | 4.210E-03 | [8](javascript:ViewExpDataObj.network_options_func(3262);) | 44 |
|  | IL-6 signaling pathway | 2.029E-03 | [7](javascript:ViewExpDataObj.network_options_func(479);) | 31 |
|  | IL-9 signaling pathway | 4.964E-03 | [7](javascript:ViewExpDataObj.network_options_func(523);) | 36 |
|  | MIF-mediated glucocorticoid regulation | 2.110E-04 | [7](javascript:ViewExpDataObj.network_options_func(2022);) | 22 |
|  | Murine NKG2D signaling | 3.115E-03 | [8](javascript:ViewExpDataObj.network_options_func(2251);) | 42 |
|  | Oncostatin M signaling via MAPK in human cells | 2.623E-04 | [9](javascript:ViewExpDataObj.network_options_func(2204);) | 37 |
|  | Oncostatin M signaling via MAPK in mouse cells | 9.001E-04 | [8](javascript:ViewExpDataObj.network_options_func(2205);) | 35 |
|  | Regulation of T cell function by CTLA-4 | 1.097E-03 | [8](javascript:ViewExpDataObj.network_options_func(3056);) | 36 |
|  | Role of HMGB1 in dendritic cell maturation and migration | 4.601E-03 | [6](javascript:ViewExpDataObj.network_options_func(6114);) | 27 |
|  | Role of integrins in NK cells cytotoxicity | 1.595E-03 | [8](javascript:ViewExpDataObj.network_options_func(2228);) | 38 |
|  | Signaling pathway mediated by IL-6 and IL-1 | 1.655E-03 | [7](javascript:ViewExpDataObj.network_options_func(478);) | 30 |
|  | Th17 cell differentiation | 4.212E-03 | [7](javascript:ViewExpDataObj.network_options_func(3268);) | 35 |
| Chemotaxis | CXCR4 signaling pathway | 1.300E-04 | [9](javascript:ViewExpDataObj.network_options_func(617);) | 34 |
| Pathogenesis of obesity | Metabolic syndrome X (Scheme) | 1.400E-03 | [5](javascript:ViewExpDataObj.network_options_func(5952);) | 15 |
|  | Pioglitazone and Rosiglitazone in treatment of type 2 diabetes and metabolic syndrome X | 1.258E-06 | [10](javascript:ViewExpDataObj.network_options_func(6251);) | 26 |
|  | Processes in pathogenesis of Diabetes mellitus II, Insulinoresistance and Obesity | 4.583E-04 | [6](javascript:ViewExpDataObj.network_options_func(11157);) | 18 |
|  | Putative pathways for stimulation of fat cell differentiation by Bisphenol A | 4.719E-04 | [8](javascript:ViewExpDataObj.network_options_func(5030);) | 32 |
|  | Role of adipose tissue hypoxia in obesity and type 2 diabetes | 6.309E-08 | [12](javascript:ViewExpDataObj.network_options_func(4724);) | 30 |
|  | Role of IL-6 in obesity and type 2 diabetes in adipocytes | 2.465E-03 | [7](javascript:ViewExpDataObj.network_options_func(4911);) | 32 |
|  | Role of Leptin in regulation of eating behavior in obesity | 2.878E-04 | [7](javascript:ViewExpDataObj.network_options_func(5218);) | 23 |
|  | Role of Nicotine-induced Leptin resistance in hypothalamus in development of obesity | 3.050E-03 | [6](javascript:ViewExpDataObj.network_options_func(5060);) | 25 |
|  | Role of TNF-alpha, IL-1beta and IL-6 in development of obesity and type 2 diabetes in liver | 3.900E-04 | [10](javascript:ViewExpDataObj.network_options_func(4910);) | 47 |
|  | Fenofibrate in treatment of type 2 diabetes and metabolic syndrome X | 8.244E-06 | [9](javascript:ViewExpDataObj.network_options_func(6252);) | 25 |
|  | GLP-1 in beta cell apoptosis in type 2 diabetes | 1.959E-03 | [9](javascript:ViewExpDataObj.network_options_func(5583);) | 48 |
|  | GLP-1 in inhibition of beta cell proliferation and function in type 2 diabetes | 2.603E-05 | [11](javascript:ViewExpDataObj.network_options_func(5582);) | 42 |
|  | Adiponectin in pathogenesis of type 2 diabetes | 3.253E-05 | [9](javascript:ViewExpDataObj.network_options_func(5951);) | 29 |
|  | Selective Insulin resistance in type 2 diabetes in liver | 7.746E-05 | [9](javascript:ViewExpDataObj.network_options_func(5349);) | 32 |
|  | Chemokines in inflammation in adipose tissue and liver in obesity, type 2 diabetes and metabolic syndrome X | 3.195E-06 | [13](javascript:ViewExpDataObj.network_options_func(13015);) | 48 |
|  | Chylomicron dyslipidemia in type 2 diabetes and metabolic syndrome X | 2.256E-03 | [8](javascript:ViewExpDataObj.network_options_func(13016);) | 40 |
| Signal transduction | IP3 signaling | 2.275E-03 | [9](javascript:ViewExpDataObj.network_options_func(557);) | 49 |
|  | JNK pathway | 3.115E-03 | [8](javascript:ViewExpDataObj.network_options_func(526);) | 42 |
|  | PTEN pathway | 1.884E-06 | [13](javascript:ViewExpDataObj.network_options_func(676);) | 46 |
|  | Some pathways of EMT in cancer cells | 3.027E-03 | [9](javascript:ViewExpDataObj.network_options_func(3017);) | 51 |
|  | Androgen Receptor nuclear signaling | 1.429E-06 | [13](javascript:ViewExpDataObj.network_options_func(2202);) | 45 |
|  | PPAR Pathway | 7.539E-05 | [13](javascript:ViewExpDataObj.network_options_func(546);) | 63 |
|  | Receptor-mediated HIF regulation | 4.012E-04 | [9](javascript:ViewExpDataObj.network_options_func(416);) | 39 |
|  | Role of AP-1 in regulation of cellular metabolism | 5.809E-05 | [10](javascript:ViewExpDataObj.network_options_func(434);) | 38 |
|  | Role of VDR in regulation of genes involved in osteoporosis | 8.988E-04 | [11](javascript:ViewExpDataObj.network_options_func(669);) | 61 |
|  | Regulation of EIF2 activity | 7.398E-05 | [10](javascript:ViewExpDataObj.network_options_func(497);) | 39 |
|  | Regulation of EIF4F activity | 5.444E-05 | [12](javascript:ViewExpDataObj.network_options_func(496);) | 53 |
|  | Non-genomic (rapid) action of Androgen Receptor | 4.905E-04 | [9](javascript:ViewExpDataObj.network_options_func(2203);) | 40 |
| Regulation of metabolism | **FXR-regulated cholesterol and bile acids cellular transport** | **7.545E-08** | [**14**](javascript:ViewExpDataObj.network_options_func(3091);) | **42** |
|  | FXR-dependent negative-feedback regulation of bile acids concentration | 2.029E-03 | [7](javascript:ViewExpDataObj.network_options_func(3090);) | 31 |
|  | Insulin regulation of glycogen metabolism | 1.648E-03 | [10](javascript:ViewExpDataObj.network_options_func(725);) | 56 |
|  | PPAR regulation of lipid metabolism | 2.603E-05 | [11](javascript:ViewExpDataObj.network_options_func(632);) | 42 |
|  | **RXR-dependent regulation of lipid metabolism via PPAR, RAR and VDR** | **2.921E-04** | [**8**](javascript:ViewExpDataObj.network_options_func(413);) | **30** |
|  | **Bile acids regulation of glucose and lipid metabolism via FXR** | **1.093E-09** | [**15**](javascript:ViewExpDataObj.network_options_func(3089);) | **37** |
|  | Role of Adiponectin in regulation of metabolism | 8.610E-04 | [9](javascript:ViewExpDataObj.network_options_func(4733);) | 43 |
|  | Fatty Acid Omega Oxidation | 3.549E-03 | [7](javascript:ViewExpDataObj.network_options_func(908);) | 34 |
| Protein folding and processing | Angiotensin system maturation \ Human version | 5.440E-06 | [12](javascript:ViewExpDataObj.network_options_func(2647);) | 43 |
|  | Angiotensin system maturation \ Rodent version | 1.874E-05 | [12](javascript:ViewExpDataObj.network_options_func(2648);) | 48 |
