# Supplementary figures and images for: Treatment of mouse liver slices with cholestatic hepatotoxicants results in down-regulation of Fxr and its target genes
Source: BMC Med Genomics. 2013 Oct 10;6:39. doi: 10.1186/1755-8794-6-39 (PMC3852711; doi:10.1186/1755-8794-6-39)

## Slide 1
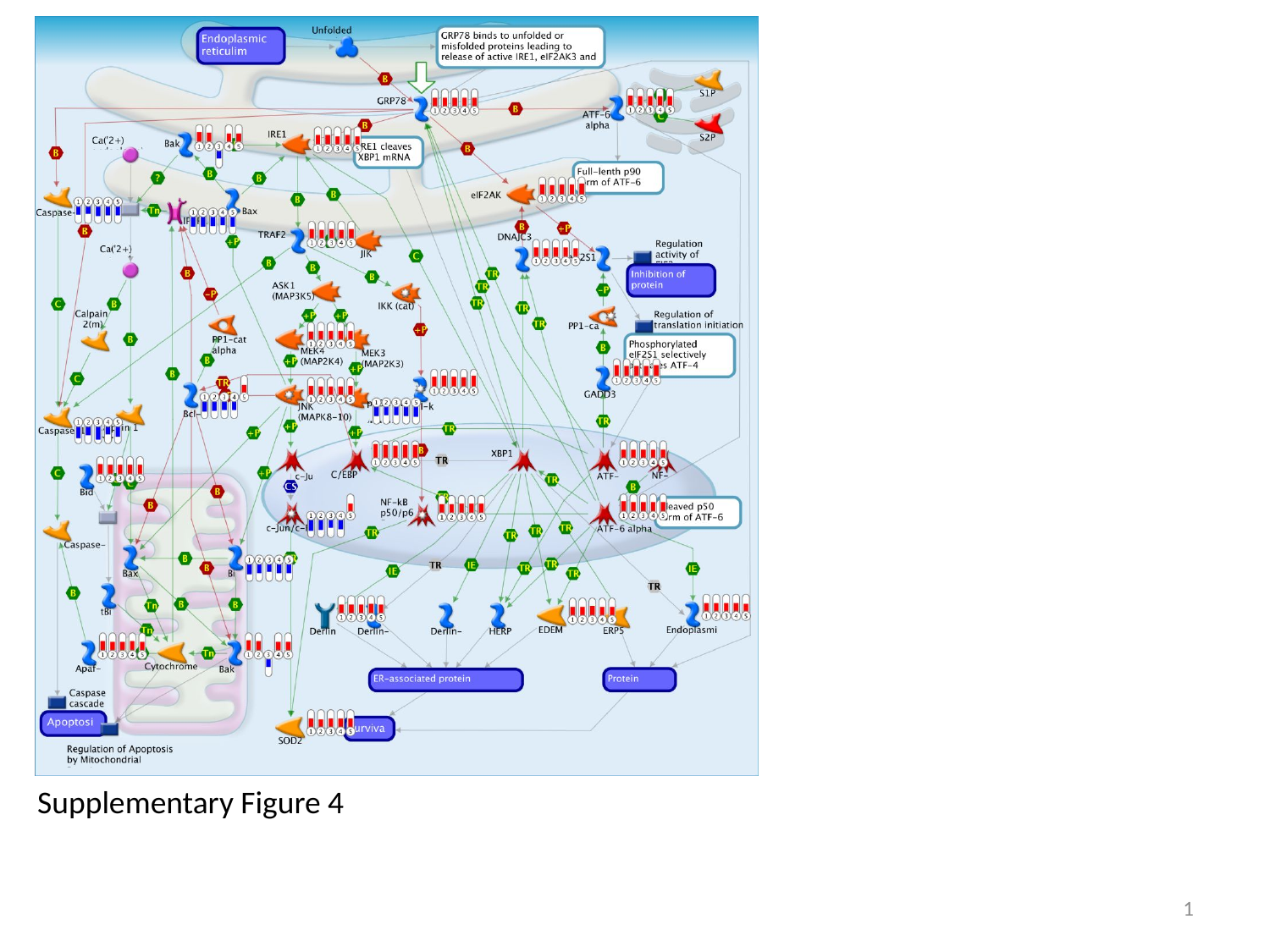

Supplementary Figure 4
1

Supplement: Additional file 7: Figure S4 — MetaCore pathway analysis in CsA treated slices: Endoplasmic reticulum stress response pathway. Cyclosporin A (CsA) significantly affected Endoplasmic reticulum stress response pathway (p < 0.005). Blue (down-regulation) and red (up-regulation) bars indicate significantly affected genes. The numbers 1–5 represent fold change (treatment vs. control) of gene expression of five independent experiments (i.e. liver slices isolated from 5 mice). [file 1755-8794-6-39-S7.pptx]

## Slide 1
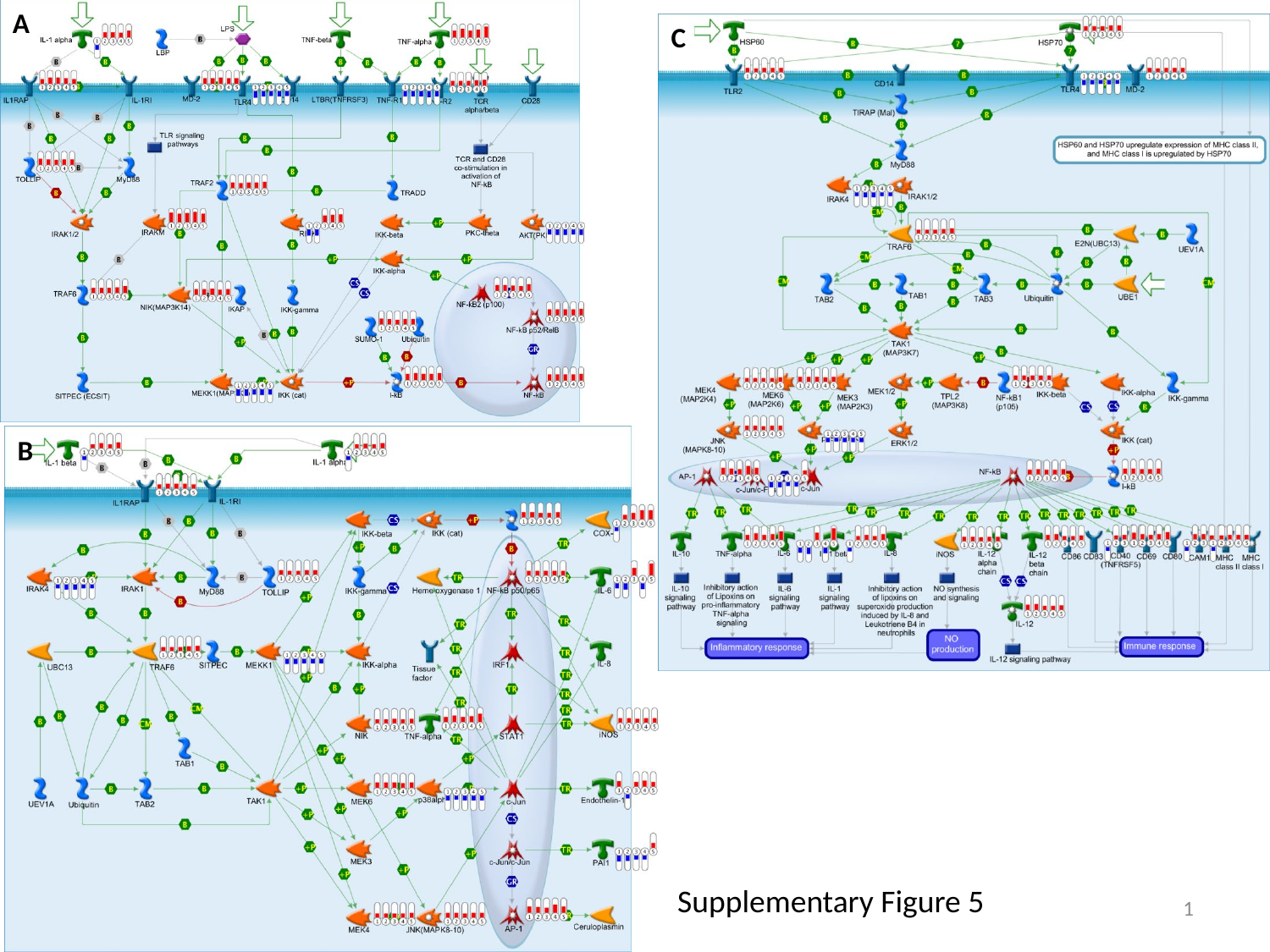

A
C
B
Supplementary Figure 5
1

Supplement: Additional file 8: Figure S5 — MetaCore pathway analysis in CsA treated slices: Nf-κB signaling Il-1 signaling pathway, and Hsp60 and Hsp70 Tlr signaling pathways. PCLS treated with CsA displayed significant (p < 0.005) up regulation of NF-κB signaling (A, Il-1 signaling pathway (B), and Hsp60 and Hsp70 Tlr signaling pathways (C). Blue (down-regulation) and red (up-regulation) bars indicate significantly affected genes. The numbers 1–5 represent fold change (treatment vs. control) of gene expression of five independent experiments (i.e. liver slices isolated from 5 mice). [file 1755-8794-6-39-S8.pptx]

## Slide 1
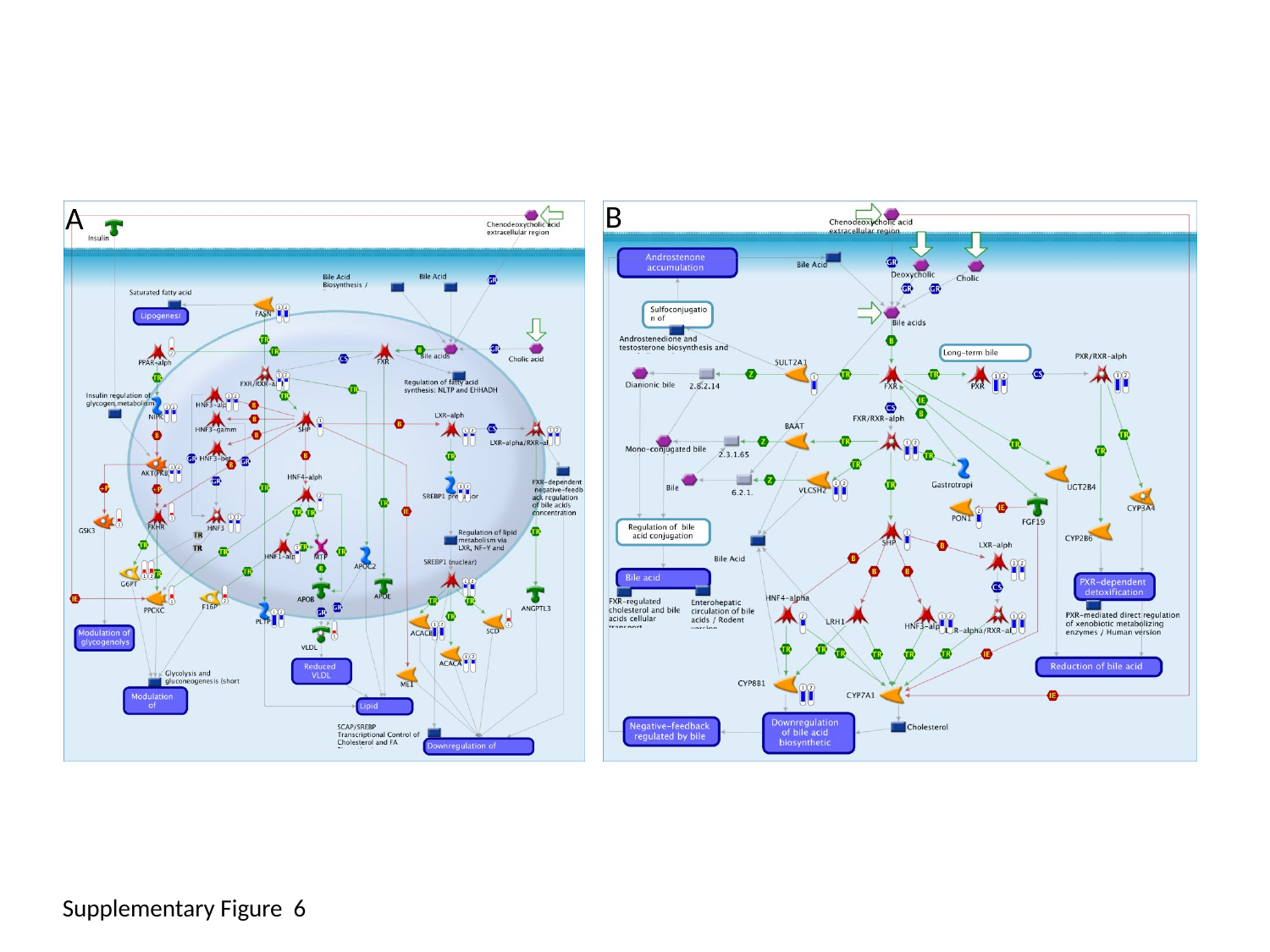

B
A
Supplementary Figure 6

Supplement: Additional file 10: Figure S6 — A-B. Effects of cholestasis on the expression of genes involved in Fxr-regulated pathways related to lipid and bile acids metabolism in human liver biopsies. Both biliary atresia and intrahepatic cholestasis significantly down-regulated Bile acids regulation of glucose and lipid metabolism via Fxr (A), and Fxr-dependent negative-feedback regulation of bile acids concentration (B). Blue and red bars indicate down-and up-regulation respectively of significantly affected genes. The bar numbers 1 and 2 indicate liver biopsies obtained from patients suffering from biliary atresia and intrahepatic cholestasis, respectively. Each bar represents average fold change of gene expression (disease vs. control) in liver biopsies. For an explanation of the MetaCore symbols is referred to http://pathwaymaps.com/pdf/MC_legend.pdf. [file 1755-8794-6-39-S10.pptx]

## Slide 1
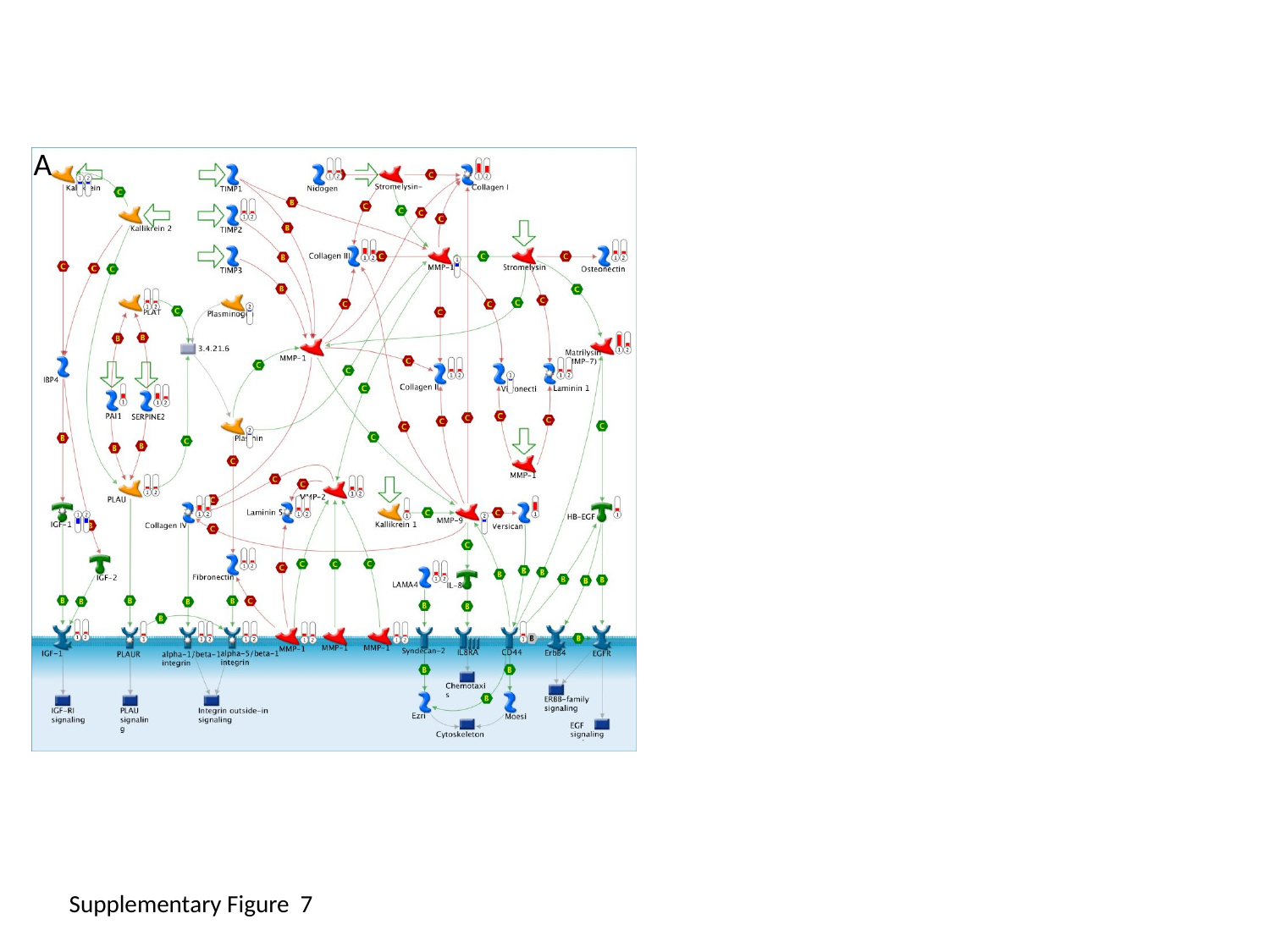

A
Supplementary Figure 7

Supplement: Additional file 11: Figure S7 — A. Cell adhesion ECM remodelling pathway is affected in liver biopsies of patients suffering from cholestasis. In two types of human cholestasis, biliary atresia (bar 1) and intrahepatic cholestasis (bar 2) both Cell adhesion_ECM remodelling pathway (p < 0.005) (A) was affected. Explanation of the bars is given in the legend of Additional file 10: Figure S6. [file 1755-8794-6-39-S11.pptx]
