# Supplementary material for: Treatment of mouse liver slices with cholestatic hepatotoxicants results in down-regulation of Fxr and its target genes
Source: BMC Med Genomics. 2013 Oct 10;6:39. doi: 10.1186/1755-8794-6-39 (PMC3852711; doi:10.1186/1755-8794-6-39)
Supplement: Additional file 9: Table S4 — Functions of genes tested by q-PCR, source GeneCards http://www.genecards.org. [file 1755-8794-6-39-S9.doc]

**Supplementary Table 4. Functions of genes tested by q-PCR, source GeneCards http://www.genecards.org.**

| Gene | Function |
| --- | --- |
| Baat | The protein encoded by this gene catalyzes the transfer of the bile acid moiety from the acyl-CoA thioester to either glycine or taurine, the second step in the formation of bile acid-amino acid conjugates which serve as detergents in the gastrointestinal tract. |
| Abcg8 | The protein encoded by this gene functions as a half-transporter to limit intestinal absorption and promote biliary excretion of sterols. |
| Abcg5 | The protein encoded by this gene functions as a half-transporter to limit intestinal absorption and promote biliary excretion of sterols. |
| Klf15 | KLF15 is increased by fasting and decreased by feeding. Plays a role in regulation of energy metabolism and gluconeogenesis. |
